# An Information-Theoretic View of Mixed-Delay Traffic in 5G and 6G

**DOI:** 10.3390/e24050637

**Published:** 2022-04-30

**Authors:** Homa Nikbakht, Michèle Wigger, Malcolm Egan, Shlomo Shamai (Shitz), Jean-Marie Gorce, H. Vincent Poor

**Affiliations:** 1 INRIA, INSA, CITI, Université de Lyon, EA3720, 69621 Villeurbanne, France; 2 LTCI, Télécom Paris, Institut Polytechnique de Paris, 91120 Palaiseau, France; 3 Department of Electrical and Computer Engineering, Technion–IIT, Haifa 3200003, Israel; 4 School of Engineering and Applied Science, Princeton University, Princeton, NJ 08544, USA

**Keywords:** mixed-delay constraints, URLLC, eMBB

## Abstract

Fifth generation mobile communication systems (5G) have to accommodate both Ultra-Reliable Low-Latency Communication (URLLC) and enhanced Mobile Broadband (eMBB) services. While eMBB applications support high data rates, URLLC services aim at guaranteeing low-latencies and high-reliabilities. eMBB and URLLC services are scheduled on the same frequency band, where the different latency requirements of the communications render their coexistence challenging. In this survey, we review, from an information theoretic perspective, coding schemes that simultaneously accommodate URLLC and eMBB transmissions and show that they outperform traditional scheduling approaches. Various communication scenarios are considered, including point-to-point channels, broadcast channels, interference networks, cellular models, and cloud radio access networks (C-RANs). The main focus is on the set of rate pairs that can simultaneously be achieved for URLLC and eMBB messages, which captures well the tension between the two types of communications. We also discuss finite-blocklength results where the measure of interest is the set of error probability pairs that can simultaneously be achieved in the two communication regimes.

## 1. Introduction

Modern communication networks serve a range of applications with heterogenous characteristics. Indeed, 5G and proposed 6G wireless mobile cellular networks are expected to serve a diverse set of applications including telephony, video-streaming, online gaming, time-critical control for transportation or remote surgery, or massive machine-type applications for sensor networks in the Internet of Things (IoT) [1]. These applications differ in terms of both reliability and latency requirements. A key example is when *Ultra-Reliable Low-Latency Communication (URLLC)* and *enhanced Mobile Broadband (eMBB)* [2] applications utilize the same time-frequency resource blocks.

URLLC is designed to ensure 99.99% reliability at a maximum end-to-end delay of no more than one millisecond [3,4,5,6,7,8,9,10]. It is thus suited for delay- and mission-critical applications such as remote surgery, control of manufacturing sites, or communication to and from autonomous vehicles. On the other hand, eMBB is most prominently used for video streaming and other applications with less stringent delay tolerances [2].

**Figure 1:**
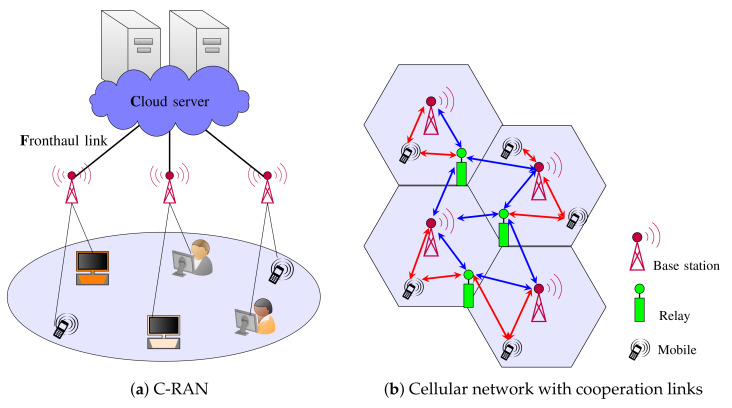
Infrastructures that allow the mitigation of interference.

In 5G and proposed 6G systems, URLLC and eMBB users are allocated *network slices*, which correspond to resources within the radio access network. A key challenge is how to design resource allocation and coding schemes given that URLLC and eMBB slices have very different delay requirements. As such, the network must support *mixed delay traffic*. This challenge is further complicated when the radio access network exploits advanced architectures, such as *cloud radio access networks (C-RANs)* [11,12] (illustrated in Figure 1a) or cooperative networks (illustrated in Figure 1b).

One standard approach is to use smart scheduling and resource allocation algorithms, which interrupt eMBB transmissions to send URLLC data. Various scheduling algorithms have been proposed, which exploit machine learning techniques [13,14,15,16] (including deep learning [17,18]) and intelligent reflective surfaces [19] to improve performance.

**Figure 2:**
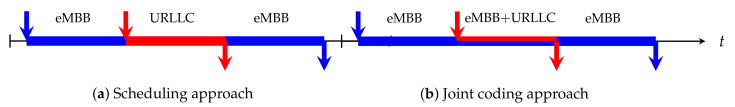
URLLC and eMBB transmissions: (**a**) Scheduling approach, (**b**) Joint coding approach.

Nevertheless, an *information theoretic perspective* suggests that performance can be further improved via advanced *joint* coding schemes, which account for the mixed delay requirements of URLLC and eMBB slices. Indeed, by introducing joint coding schemes, data from both URLLC and eMBB slices can be simultaneously transmitted in the same resource block, as illustrated in Figure 2. A key issue is that interference is introduced not only by multiple users within the same time-frequency resource, but also from data from each slice transmitted by the *same* user. Nevertheless, as we show in this survey, by using appropriate joint coding techniques, the presence of interference does not necessarily lead to reductions in performance.

In this survey, we overview recent work on joint coding with mixed delay traffic arising from URLLC and eMBB slices in network architectures ranging from point-to-point to C-RANs.

### 1.1. Related Works

While in this survey we focus on communication scenarios with different network slices that have different delay constraints, information-theorists have also studied related scenarios with other types of heterogeneous communication requirements. The works most closely related to mixed-delay are [20,21,22]. Specifically, [20] studies a scenario where two messages are transmitted over a broadcast channel, but only one of them can profit from the cooperation link between the two receivers. The other message has to be decoded directly based on the legitimate receiver’s channel outputs, without cooperation from the other receiver. The motivation in [20] to study such a system was to design a robust communication scheme where the receivers can reliably decode two messages when the cooperation link between the receivers is present, while still being able to reliably decode a single message in case the cooperation link fails. A different interpretation, but with the same mathematical model, is to say that one of the messages needs to be decoded immediately without waiting for the cooperation message from the other receiver, while the other message can tolerate more delay and therefore be decoded also based on the cooperation message. In this sense, the model [20] well suits also a mixed-delay communication scenario with URLLC and eMBB slices as considered in this survey. The works in [21,22] study a scenario with different reliability criteria of two slices, as also characteristic for URLLC and eMBB slices. In particular, [21] imposes the constraint that the data from one slice has to be decoded even under an adversarial attack model (the arbitrarily varying channel [23,24,25]) whereas the data from the other slice only has to be decoded in the likely event that the channel exhibits an expected behavior.

### 1.2. Related Surveys and Contributions

A number of surveys, summarized in Table 1, have recently appeared covering varying aspects of coding, resource allocation, and architecture design in 5G and beyond. The surveys in [11,26,27] have focused on system level approaches in order to support network slicing, but do not consider aspects related to coding.

On the other hand, the surveys in [2,28,29,30,32,33,34,35,36] focus on communication theoretic aspects of 5G and future 6G systems. In particular, [2,28,29,32] consider various resource allocation techniques and specifically [2] treates resource allocation for eMBB and URLLC slices. The surveys in [33,34,35,36] focus on non-orthogonal multiple access (NOMA) schemes based on successive interference cancellation for multiple access networks, while the survey [30] overviews the benefits of rate-splitting techniques on the multiple-access channel.

Despite the importance of joint coding schemes with mixed delay traffic, there has not been a comprehensive survey on this topic. This survey aims to fill this gap by highlighting how joint coding can improve performance of standard scheduling schemes, drawing on fundamental insights from an information theoretic analysis of the network.

The main contributions in this survey are summarized as follows:

(i) An overview of interference mitigation techniques drawn from information theory, with a focus on superposition coding, dirty paper coding, and coordinated multi point transmission and reception.
(ii) A summary of joint coding schemes and recent results on their performance for mixed delay traffic in
  (a) point-to-point networks;
  (b) broadcast networks;
  (c) cooperative networks;
  (d) C-RANs.
(iii) A discussion of open problems in the design of joint coding schemes for mixed delay traffic.

### 1.3. Outline of the Survey

This survey article is organized as follows. In Section 2 we review known interference mitigation techniques such as superposition coding, dirty-paper coding, and Coordinated Multi-Point (CoMP) transmission and reception. For a more thorough discussion of these tools, we refer to the original articles or standard textbooks [37,38]. We then continue in Section 3 to discuss integrated transmission of URLLC and eMBB messages on P2P channels with a single transmitter and a single receiver, followed by Section 4 which discusses extensions to multi-receiver broadcast channels (BC). Section 5 and Section 6 consider mixed-delay transmissions over cooperative cellular interference networks and C-RANs. The survey is concluded with a summary and outlook section in Section 7.

*Notation:* Throughout the survey, we abbreviate *transmitter* and *receiver* by *Tx* and *Rx*. For *independent and identically distributed* we use *i.i.d.* Random variables are denoted using upper case letters, and realizations thereof by lower case letter, e.g., *X* and *x*. Random vectors are denoted with uppercase bold symbols. Fixed constants are often written with sans-serif font, for example K,P,Q or using Greek letters, for example ρ and α. To follow standard notation we however use *n* to denote the blocklength of transmission. For any positive integer K we use the short-hand notation [K]={1,…,K}. Channels are assumed to be real-valued, extensions to complex channels with independent real and complex components are straightforward. In this sense, $N(0,\sigma^{2})$ denotes the real centered Gaussian distribution of variance $\sigma^{2}$. We also use the usual shorthand notation $Y^{n}=\left(Y_{1},\ldots ,Y_{n}\right)$.

## 2. Mixed Delay Traffic and Interference Mitigation

### 2.1. Coding and Delay

The primary goal of a communications network is to reliably send one or more messages $M_{i}\in \left\{1,\ldots ,M_{i}\right\}$ from one or more Txs to one or more Rxs. To do so, each Tx *encodes* the different messages it wishes to send into a waveform $x_{k}^{n}$, which corresponds to the physical signal sent over the network. In a scenario with *homogeneous delay constraints* i.e., where all messages are sent over the same blocklength *n*, a Tx *k* encodes its messages $\left\{M_{i}:i\in T_{k}\right\}$, where $T_{k}$ collects the indices of all messages sent by Tx *k*, into the codeword $x_{k}^{n}\left(\left\{M_{i}:i\in T_{k}\right\}\right)$ using a joint encoding function $f_{k}:\left\{1,\ldots ,M_{i_{k}}\right\}\times \cdots \times \left\{1,\ldots ,M_{i_{k+1}-1}\right\}\to R^{n}$. After receiving the corresponding output symbols $y_{k}^{n}$, Rx *k* produces a guess $\left\{{\hat{M}}_{i}:i\in R_{k}\right\}$ for each of its desired messages $\left\{M_{i}:i\in R_{k}\right\}$, where $R_{k}$ collects the indices of the messages intended for Rx *k*, by applying a decoding function $g_{k}:R^{n}\to \left\{1,\ldots ,M_{i_{k}}\right\}\times \cdots \times \left\{1,\ldots ,M_{i_{k+1}-1}\right\}$ to its observed outputs $y_{k}^{n}$.

A more complicated scenario typically arises in the mixed-delay scenarios we consider in this survey, because the various messages are created at different times and have different decoding delays. In this case, each message is assigned a creation time $a_{i}$ and a latest possible decoding time $d_{i}$. As a consequence, a Tx *k* produces its inputs $x_{k}^{n}$ using per-symbol encoding functions ${\left\{f_{k,t}\right\}}_{t}$, where at time-*t* the function $f_{k,t}$ maps all its previously created messages to an input symbol:

(1) $$x_{k,t}=f_{k,t}\left(\left\{M_{i}:i\in T_{k},a_{i}\leq t\right\}\right).$$

Rx *k* decodes any of its intended messages $\left\{M_{i}:i\in R_{k}\right\}$ by applying the decoding function $g_{i}$ to the first $d_{i}$ channel outputs $y_{k}^{d_{i}}$. The decoding function that produces the message guess ${\hat{M}}_{i}$ is thus of the form $g_{i}:R^{d_{i}}\to \left\{1,\ldots ,M_{i}\right\}$.

Associated with the described mixed-delay encodings are two key parameters:

(i) the length of each codeword, $n_{i}=d_{i}-a_{i}$;
(ii) and the *rate*, defined by
  (2) $$\begin{array}{c} R_{i}=\frac{\log M_{i}}{n_{i}}. \end{array}$$

In multi-hop scenarios such as experienced in C-RANs or cooperative networks, transmission delay not only depends on the blocklength of communication, but also on the delay introduced from the communication over the addition hops. For example, in the uplink of C-RANs, the total delay experienced for the transmission of a messages is formed by:

- the communication delay over the network from the mobile users to the BSs;
- the processing time of the compression at the BSs as well as the communication delay over the fronthaul links to the cloud processor;
- the decoding processing time at the cloud processor.

In mixed-delay networks, the additional delay introduced by the compression at the BSs and the fronthaul communication might exceed the latest allowed decoding time $d_{i}$ for certain messages $M_{i}$, which thus have to be directly decoded at the BSs. A similar situation is also encountered in the downlink of C-RANs, where URLLC messages should directly be encoded at the BSs and not at the cloud processor so as to avoid the delay introduced by the additional communication hop over the fronthaul link. In the same way, URLLC messages transmitted in cooperative interference networks cannot support the additional communication hops required to establish cooperation between Txs or Rxs. In these networks, the cooperative communication at the Tx side thus can only depend on eMBB messages and the cooperative communication at the Rx side can only serve decoding of eMBB messages. We will provide a more detailed model for the encoding and decoding of mixed-delay messages in Section 5 ahead. A main assumption in our model will be that the communication over the interference network is sufficiently short so that also URLLC messages can tolerate the introduced delay.

As we will overview in this survey, mixed delay constraints require careful design of the joint coding schemes, in particular to mitigate the interference caused by the different slices. In the remainder of this section, we summarize key information-theoretic interference mitigation techniques.

### 2.2. Superposition Coding

**Figure 3:**
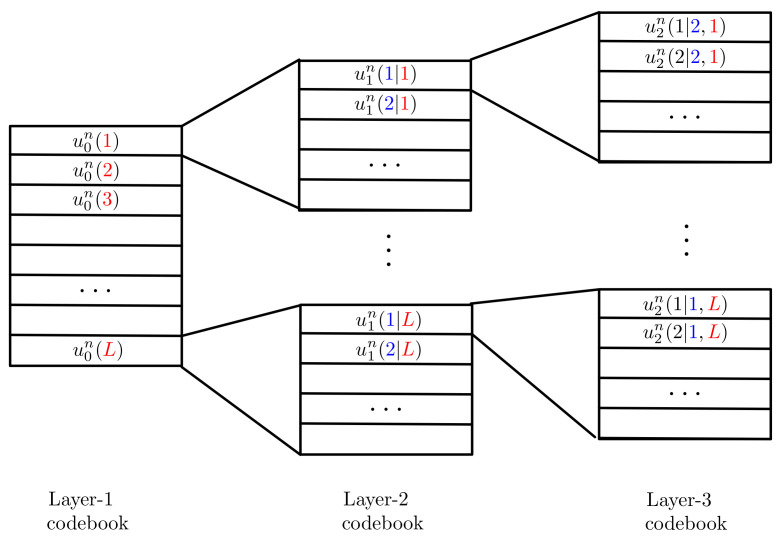
Superposition code with 3 layers.

Superposition coding [37,39] was first proposed in the context of broadcast communication. It can be used to send multiple messages to one or more receivers. The main technical feature is that the different messages are encoded into the different layers of a so called superposition code, illustrated in Figure 3 for a code example with three layers. The entries of the layer-1 code are drawn i.i.d. according to a chosen distribution $P_{U_{0}}$. *For each layer-1 codeword $u_{0}^{\left(n\right)}\left(\ell \right)$ a new layer-2 codebook is chosen.* The entries of the layer-2 codewords are drawn independent of each other and the *i*-th entry follows a conditional distribution $P_{U_{1}|U_{0}}$ given the *i*-th entry of codeword $u_{0}^{\left(n\right)}\left(\ell \right)$. For each layer-2 codeword, a new layer-3 codebook is chosen. Entries of this codebook are again independently of each other and drawn according to a conditional distribution $P_{U_{2}|U_{1}}$ given the entries in the corresponding layer-2 codeword.

In a superposition code, each message is not only protected by its corresponding layer, but also by all underlying layers. Given the structure of the code, any receiver that is interested in decoding a given layer also has to decode *all previous layers*, typically in a joint manner. Upper layers are not decoded and cause additional disturbance (noise) on the decoding of lower layers.

Given the described decoding order, in the context of mixed-delay traffic it is possible to send URLLC data on lower layers and eMBB on upper layers but not the other way around, because URLLC messages have to be decoded first, prior to decoding eMBB messages.

A simpler alternative to superposition coding is to encode each message using an independent codebook and to combine the chosen codewords, by means of a predefined mapping, to form the sequence of channel inputs. In particular, for Gaussian channels, messages are encoded into Gaussian codewords and the sum of these codewords is transmitted over the channel. The advantage of this method is that no layering-order of the codewords has to be established a priori. This is particularly convenient in fading channels where the exact channel statistics are not known at time of encoding, and the receiver can decide on the layers to decode after having estimated the realization of the channel. In this sense, the simpler alternative can allow for increased *expected rates* over slowly fading channels where the channel variations are limited over the duration of a single codeword. As we shall see, this approach is also highly beneficial for joint transmission of URLLC and eMBB messages where the fading is almost constant over the duration of an URLLC communication but varies significantly during the transmission of eMBB messages.

### 2.3. Dirty-Paper Coding (DPC)

If interference is known at a Tx before communication starts, the Tx can mitigate this interference through *Dirty Paper Coding* [40,41,42] and achieve the full capacity of the channel without interference. To illustrate, consider the Gaussian interference channel

(3) $$Y^{n}=X^{n}+W^{n}+Z^{n},$$

where $Z^{n}$ is an i.i.d. standard Gaussian noise sequence and $W^{n}$ is memoryless interference sequence, with each component zero-mean Gaussian of power Q. If $W^{n}$ is unknown to both the Tx and the Rx, the interference simply acts as additional noise, and the capacity of the channel equals $\frac{1}{2}\log\left(1+\frac{P}{1+Q}\right)$. If the Rx knows $W^{n}$, then it can subtract this interference from the outputs and as a consequence the capacity of the channel is the same as without interference, i.e., $\frac{1}{2}\log\left(1+P\right)$. Costa [40] showed that when $W^{n}$ is unknown to the Rx but known to the Tx even before the communication starts, then the capacity of the channel is also equal to the interference-free capacity $\frac{1}{2}\log\left(1+P\right)$. The coding scheme achieving this performance was termed dirty-paper coding (DPC) and is described in the following. (For an analysis see [37,40]).

Define the parameter $\alpha :=\frac{P}{1+Q}$ and the random variable U=X+αW where W∼N(0,Q) and X∼N(0,P) independent of each other. It can be verified that

(4) $$\begin{array}{ccc} I(U;Y) & = & \frac{1}{2}\log\left(\frac{\left(P+Q+1\right)\left(P+\alpha^{2}Q\right)}{PQ{\left(1-\alpha \right)}^{2}+\left(P+\alpha^{2}Q\right)}\right) \end{array}$$

(5) $$\begin{array}{ccc} I(U;W) & = & \frac{1}{2}\log\left(\frac{P+\alpha^{2}Q}{P}\right) \end{array}$$

(6) $$\begin{array}{ccc} I(U;Y)-I(U;W) & = & \frac{1}{2}\log\left(\frac{P(P+Q+1)}{PQ{\left(1-\alpha \right)}^{2}+\left(P+\alpha^{2}Q\right)}\right)=\frac{1}{2}\log\left(1+P\right)Q \end{array}$$

Fix ϵ>0 arbitrary small. For each $m\in \left[2^{n(I(U;Y)-I(U;W)-\epsilon )}\right]$ generate a bin with $2^{n(I(U;W)+\epsilon /2)}$ codewords $\left\{U^{n}\left(j,m\right):j\in \left[2^{n(I(U;W)+\epsilon /2)}\right]\right\}$ by picking each component of each codeword i.i.d. according to $N\left(0,P+\alpha^{2}Q\right)$. Reveal the codebook consisting of all $2^{n(I(U;Y)-I(U;W)-\epsilon )}$ bins to the Rx and the Tx.

*Encoding:* To encode a message M=m, the encoder looks for a codeword $U^{n}\left(j,m\right)$ in bin *m* that is jointly typical [37] (i.e., has approximately the correct joint empirical distribution) with the interference sequence $W^{n}$. The Tx then forms $X^{n}=U^{n}\left(j^{*},m\right)-\alpha W^{n}$, where $j^{*}$ indicates the chosen index, and send this sequence $X^{n}$ over the channel. Note that the Tx declares an error if no codeword $U^{n}\left(j,m\right)$ in bin *m* is jointly typical with the interference $W^{n}$.

*Decoding:* After observing the sequence $Y^{n}$, the Rx looks for a codeword $U^{n}\left(j,m\right)$ that is jointly typical with $Y^{n}$. If a single such codeword exists, the Rx declares $\hat{M}=m$, otherwise it declares an error.

It can be shown that with probability tending to 1 as the blocklength n→∞, the only codeword that is jointly typical with $Y^{n}$ is codeword $U^{n}\left(j^{*},m\right)$ which was selected at the transmitter. The Rx thus not only recovers the correct message $\hat{M}=M$ with high probability, but can also reconstruct the transmitted codeword $U^{n}\left(j^{*},m\right)$ with high probability.

### 2.4. Coordinated Multi Point (CoMP)

*Coordinated multi-point (CoMP)* refers to a wide set of techniques that enable either a set of distributed Txs to jointly encode messages or a set of distributed Rxs to jointly decode messages. We will be particularly interested in scenarios where CoMP is facilitated through cooperative communication over dedicated links between transmitters or between receivers.

In the case of *CoMP transmission* [43,44,45,46], we consider a set of distributed Txs, each having one message to send, and with cooperation links between neighbouring Txs. Before communicating to the Rxs, all Txs convey their messages to a dedicated Tx, called the *master Tx*, which then jointly designs input signals for all Txs (also exploiting its available state-information) and conveys rate-distortion compressed (lossy) versions of these signals to each of the Txs. The Txs reconstruct the compressed signals and send these signals over the channel to the Rxs. If cooperation links are of sufficiently high rates, then the loss of the compression can be maintained at noise-level and does not decrease the *degrees of freedom (DoF)*, i.e., the factor in front of the logarithmic expansion of the asymptotic high-SNR sum-capacity. In this case, the interference channel is intuitively transformed into a multi-antenna single-Tx BC and the DoF is given by the minimum number of Tx and Rx antennas.

In case of *CoMP reception* [47,48,49,50], consider a set of distributed Rxs, each wishing to decode one message, and with cooperation links between neighbouring Rxs. Each Rx applies a lossy compression algorithm to its observed output signal and describes the compressed signal over the cooperation links to a dedicated Rx, called *master Rx*. This master Rx reconstructs all the compressed signals and jointly decodes all the messages, which it then sends to their intended Rxs over the cooperation links. If cooperation links are of sufficiently large rates, then lossy compression of the receive signals can be performed so that the loss is maintained at noise-level, which again has no influence on the DoF of the channel, and corresponds to the DoF of a single-Rx multi-access channel which equals the minimum number of Tx and Rx antennas.

CoMP transmission and reception can only be used to encode and decode eMBB messages, because the communication over the cooperation links induces significant delay. The delay is in fact given by twice the number of hops required on the cooperation links to reach the master Tx/Rx from any other Tx/Rx in the network times the communication duration on a single cooperation hop. Since in practical networks also eMBB communication is delay-limited, CoMP transmission and reception can be performed only on small subsets of Txs and Rxs, where the size depends on the maximum allowed number of communication hops.

## 3. Point-to-Point Communications

### 3.1. Introduction

This section focuses on P2P channels with a single Tx and a single Rx. Section 3.2 reviews the superposition coding approach over fading channels in [51,52]. This approach manages to send URLLC messages over single coherence blocks of the fading channel without suffering from a degradation due to the lack of state knowledge at the Tx, and simultaneously also sends eMBB messages over multiple coherence blocks, thus exploiting the ergodic behaviour of the channel. This approach is also known as *broadcast approach* and has been studied for a wide field of applications, see the recent survey paper [53].

Section 3.3 summarizes the results in [54,55], which analyze a similar superposition coding approach but for Gaussian channels and in the finite-blocklength regime. The analysis is based on the concept of “parallel channels” introduced in [56].

### 3.2. The Broadcast Approach over Fading Channels without Transmitter Channel State Information

This section is based on the results in [51,52,53]. Consider a P2P channel where a single Tx wishes to send both URLLC and eMBB messages over a fading channel to a single Rx. Latency requirements impose that transmission of URLLC messages spans only a single coherence time of the fading channel, but transmission of eMBB can span multiple coherence blocks and thus profit from channel diversity. In a single-antenna setup, a simple channel model capturing these constraints is as follows:

(7) $$Y_{b,t}=\sqrt{S_{b}}\cdot X_{b,t}+Z_{b,t},\;b=1,\ldots ,B,\;t=1,\ldots ,T,$$

where B denotes the number of blocks, T the channel coherence time, $\left\{S_{b}\right\}$ describe the fading power in the various coherence blocks and are assumed i.i.d. with probability distribution function (pdf) $f_{S}$ and variance 1, and $\left\{Z_{b,t}\right\}$ is a sequence of i.i.d. standard Gaussian noises. The fading power is assumed to be perfectly known at the Rx (e.g., by transmitting pilot signals at the beginning of each block based on which the Rx can estimate the fading power), but not at the Tx. Since each URLLC message can be transmitted only over a single coherence block, the channel inputs are formed as

(8) $$X_{b,t}=f_{b,t}\left(M_{b}^{\left(U\right)},M^{\left(e\right)}\right),\;b=1,\ldots ,B,\;t=1,\ldots ,T,$$

for some appropriate encoding functions $\left\{f_{b,t}\right\}$ satisfying the power constraint

(9) $$\frac{1}{BT}\sum_{b=1}^{B}\sum_{t=1}^{T}{\left|X_{b,t}\right|}^{2}\leq P,$$

and where $M_{b}^{\left(U\right)}$ indicates the URLLC message sent in block *b* and $M^{\left(e\right)}$ the single eMBB message sent over the entire *B* blocks.

In the following, messages are assumed independent of each other and uniform over message sets $M_{U}$ and $M_{e}$. In this subsection, $M_{U}=\left[2^{TR_{U}}\right]$ and $M_{e}=\left[2^{TBR_{e}}\right]$, where $R_{U}$ and $R_{e}$ denote the URLLC and eMBB rates of transmission.

After each block *b*, the Rx decodes the URLLC message $M_{b}^{\left(U\right)}$ sent in this block:

(10) $${\hat{M}}_{b}^{\left(U\right)}=g_{b}^{\left(U\right)}\left(Y_{b,1},\ldots ,Y_{b,T}\right),\;b=1,\ldots ,B,$$

and at the end of the entire communication it also decodes the eMBB message:

(11) $${\hat{M}}^{\left(e\right)}=g^{\left(e\right)}\left(Y_{1,1},\ldots ,Y_{B,T}\right),$$

for decoding functions $\left\{g_{b}^{\left(U\right)}\right\}$ and $g^{\left(e\right)}$ on appropriate domains.

In [51,52], the authors propose to encode the two message streams using simplified superposition coding where both streams are encoded into independent Gaussian codewords, which are then added up for transmission. More precisely, the URLLC message *in each block* is encoded into multiple layers so that the Rx can decode as many layers as the actual instantaneous fading power $S_{b}$ permits. (This implies also that depending on the fading realization, certain URLLC messages are not decoded and in practice have to be retransmitted in the next block.) To allow for closed-form expressions, an infinite layering approach is employed with layers that are of infinitesimally small power.

The Tx allocates total power βP to the transmission of the URLLC messages and power (1−β)P to transmit the eMBB messages. The power distribution to the different URLLC layers is described by a power density ρ(·) satisfying $\int_{u}\rho \left(u\right)du=\beta P$, where $\rho (s^{\prime })$ indicates the (infinitesimally small) power that is assigned to a given layer that is decoded whenever the fading $S_{b}\geq s^{\prime }$. The interference power stemming from non-decoded URLLC messages under state $S_{b}=s$ is then given by s·I(s) where

(12) $$I\left(s\right):=\int_{u=s}^{\infty }\rho \left(u\right)du.$$

Since URLLC messages are decoded after each block, and eMBB messages only at the end of the last block *B*, decoding of URLLC messages not only suffers from the interference of non-decoded URLLC messages, but also from the interference of eMBB messages. The power of this latter interference is equal to (1−β)P independent of the block (since the Tx has no knowledge about $\left\{S_{b}\right\}$ it cannot adapt the power).

To decode the eMBB message at the end of the last block B, the Rx first subtracts the contributions of the codewords corresponding to the decoded URLLC messages and then decodes the eMBB message based on this difference while accounting for the interference power created by all non-decoded URLLC messages, which in block *b* is given by $I(S_{b})$.

A careful analysis of the infinite-layering approach, see [52], reveals that the expected rate of the reliably decoded messages (i.e., messages decoded with error probability tending to 0 as the blocklength T→∞) can be as high as

(13) $$R^{\left(U\right)}=\int_{u=0}^{\infty }\left(1-F_{S}\left(u\right)\right)\frac{u\rho (u)}{1+u(I(u)+(1-\beta )P)}du,$$

where $F_{S}\left(\cdot \right)$ denotes the cumulative distribution function (cdf) associated with the pdf $f_{S}\left(\cdot \right)$. In the denominator of (13), the term u(I(u)+(1−β)P) indicates the interference power experienced during the decoding of URLLC messages stemming from the eMBB transmission and the non-decoded URLLC messages.

For a sufficiently large number of blocks B, the following rate is achievable for the eMBB messages:

(14) $$R^{\left(e\right)}=\int_{u=0}^{\infty }f_{S}\left(u\right)\log\left(1+\frac{(1-\beta )Pu}{1+uI(u)}\right)du.$$

Here we find uI(u) in the denominator which describes the interference power of the non-decoded URLLC messages.

Equations (13) and (14) thus determine the maximum achievable (expected) sum-rate $R^{\left(U\right)}+R^{\left(e\right)}$ in function of the URLLC interference power I(u) (notice that $\rho \left(u\right)=-\frac{d}{du}I\left(u\right)$), which is a design parameter of the scheme and can be optimized. It is shown in [52] that the optimal interference power function I(s) among all continuously differentiable functions satisfying the boundary conditions I(0)=βP and I(∞)=0 is given by:

(15) $$I^{*}\left(s\right)=\frac{1}{2}\left(\frac{-b\left(s\right)+\sqrt{b^{2}\left(s\right)-4a\left(s\right)c\left(s\right)}}{2a(s)}\right),$$

for $a\left(s\right)=sf_{S}\left(s\right)$, $b\left(s\right)=2\left(1-\beta \right)Pf_{S}\left(s\right)s^{2}-\left(1-F_{s}\left(s\right)\right)$, and $c\left(s\right)={\left(1-\beta \right)}^{2}P^{2}f_{S}\left(s\right)s^{3}$.

**Figure 4:**
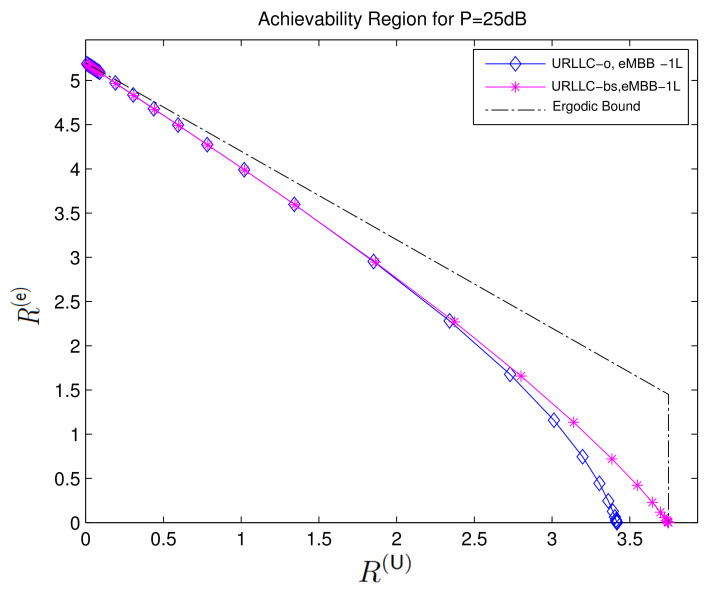
The figure illustrates the set of rate pairs $\left(R^{\left(U\right)},R^{\left(e\right)}\right)$ in function of the power allocation parameter β and of the interference powers in (15) and (16), respectively.

Figure 4 compares the sum-rate achieved for this optimal interference power $I^{*}\left(s\right)$ for different power allocation parameters β to a simple outage-based approach where ρ(s) is chosen as a dirac-function at threshold $s_{\mathrm{th}}$, i.e., when the interference power is given by the step function

(16) $$I_{o}\left(s\right)=𝟙\left\{s<s_{\mathrm{th}}\right\}.$$

Here, the optimal value for the threshold $s_{\mathrm{th}}$ can be derived analytically and is given by the solution to the following equation

(17) $$f_{S}\left(s_{\mathrm{th}}\right)\log\left(1+\beta Ps_{\mathrm{th}}\right)=\left(1-F_{S}\left(s_{\mathrm{th}}\right)\right)\frac{\beta P}{\left(1+Ps_{\mathrm{th}}\right)\left(1+\left(1-\beta \right)Ps_{\mathrm{th}}\right)}.$$

From Figure 4, we observe that for small URLLC rates $R^{\left(U\right)}$, the penalty in eMBB rates $R^{\left(e\right)}$ is small when using the suboptimal power allocation corresponding to $I_{o}\left(s\right)$ instead of the optimal allocation corresponding to $I^{*}\left(s\right)$. For larger URLLC rates, this penalty increases.

We further observe that the maximum sum-rate achieved by both power allocations decreases with increasing URLLC rates. The sum-rate for both approaches is more than 5 when $R^{\left(U\right)}=0$. For $R^{\left(U\right)}\geq 3.5$ it is around 4 under the optimal power allocation and even vanishes completely under outage power allocation leading to (16). In this high-URLLC-rate regime, the gap to the outer bound is also significant, leaving open the possibility of finding better coding schemes.

The described broadcast approach can further be improved by applying a multi-layering approach also for the transmitted eMBB message. In this approach, different eMBB layers are decoded successively, and after each eMBB decoding step, the Tx decodes further URLLC layers so as to remove their interference for the decoding of subsequent eMBB layers. This additional decoding of URLLC messages at the end of block B cannot be used to improve performance of the URLLC communication, because the admissible delay is exceeded. However, it enables an improvement in the decoding performance of eMBB messages.

Another way to improve this broadcast approach is to combine it with adaptive causal network coding. For example, the work in [57] proposes a novel layering scheme consisting of a base layer and an enhancement layer for data streaming under mixed-delay constraints. The base layer contains URLLC data and the enhancement layer contains eMBB data. In the proposed scheme, the base layer is encoded using a broadcast approach, which allows the Rx to decode the base layer (i.e., URLLC data) with minimum delay required. The enhancement layer is encoded using a priori and posteriori forward error correction so as to be able to control the throughput-delay trade-off of this communication.

### 3.3. Finite Block-Length Analysis over Gaussian Channels

This section is based on the results in [54,55]. We again consider a P2P scenario, but where communication is over a non-fading Gaussian channel

$$Y_{t}=X_{t}+Z_{t},\;t=1,2,\ldots ,$$

for $\left\{Z_{t}\right\}$ an i.i.d. standard Gaussian noise sequence.

The Tx has a single URLLC message and a single eMBB message to send to the Rx, where both messages are assumed to have strict creation times and fixed decoding deadlines. Specifically, transmission of eMBB message $M^{\left(e\right)}$ commences at time $t=a_{e}$ and decoding has to be performed at time $t=d_{e}$, while the URLLC message can be transmitted starting at time $t=a_{U}$ and has to be decoded at time $t=d_{U}$. We thus parametrize the message sets as $M_{U}=\left[2^{\left(d_{U}-a_{U}\right)R_{U}}\right]$ and $M_{e}=\left[2^{\left(d_{e}-a_{e}\right)R_{e}}\right]$. We also denote the transmission window of the eMBB message by $W_{e}$ and the transmission window of the URLLC messages by $W_{U}$:

(18) $$\begin{array}{c} W_{e}\overset{▵}{=}\left\{a_{e},\ldots ,d_{e}\right\},\;\;\;W_{U}\overset{▵}{=}\left\{a_{U},\ldots ,d_{U}\right\}. \end{array}$$

Since the URLLC delay $d_{U}-a_{U}$ is assumed shorter than the eMBB delay $d_{e}-a_{e}$, the following three situations can occur:

- Case 1: URLLC and eMBB transmissions do not overlap. i.e., $W_{U}\cap W_{e}=\emptyset$.
- Case 2: The eMBB transmission interval includes the URLLC transmission interval, i.e., $W_{U}\subset W_{e}$.
- Case 3: URLLC and eMBB transmissions overlap, but URLLC transmission is not included in eMBB transmission, i.e., $W_{e}\cap W_{U}\neq \emptyset$ and $W_{U}⊄W_{e}$.

**Figure 5:**
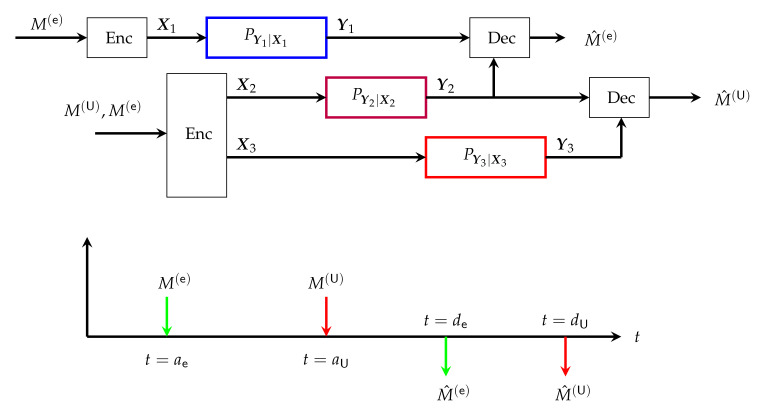
System model for transmission of URLLC and eMBB messgaes in the finite blocklength regime and under heterogeneous decoding deadline.

In Case 1 where the two messages are transmitted during independent time intervals, URLLC and eMBB transmissions can be analyzed independently based on the achievability and converse bounds in [58]. Cases 2 and 3 can be treated similarly. Here, we focus on a subcase of Case 3 where encoding starts with the eMBB message at time $t=a_{e}$ and terminates at time $t=d_{U}$ with the decoding of the URLLC message, see Figure 5. The Tx thus produces channel inputs at times $t\in \{a_{e},\ldots ,d_{U}\}$ as follows:

(19) $$\begin{array}{c} X_{t}=\left\{\begin{array}{cc} f_{t}\left(M^{\left(e\right)}\right), & \;t\in \{a_{e},\ldots ,a_{U}-1\}\\ \psi_{t}\left(M^{\left(e\right)},M^{\left(U\right)}\right), & \;t\in \{a_{U},\ldots ,d_{e}\}\\ \phi_{t}\left(M^{\left(U\right)}\right), & \;t\in \{d_{e}+1,\ldots ,d_{U}\}, \end{array}\right. \end{array}$$

where $\left\{f_{t}\right\},\left\{\psi_{t}\right\},\left\{\phi_{t}\right\}$ are appropriate encoding functions. Note that the Tx does not know the URLLC message before time $t=a_{U}$ and therefore channel inputs prior to time $t=a_{U}$ cannot depend on $M^{\left(U\right)}$. It can also be assumed that channel inputs after the eMBB decoding time $d_{e}$ de not depend on the eMBB message $M^{\left(e\right)}$. One can therefore think of the transmission taking place over three parallel channels, with respective blocklengths

(20) $$n_{1}≜a_{U}-a_{e},\;n_{2}≜d_{e}-a_{U}+1,\;\mathrm{and}\;n_{3}≜d_{U}-d_{e},$$

where the first channel consists of channel uses $\left\{a_{e},\ldots ,a_{U}-1\right\}$ and incorporates only eMBB transmission; the second channel consists of channel uses $\left\{a_{U},\ldots ,d_{e}\right\}$ and incorporates *joint transmission* of URLLC and eMBB messages; and the third channel consists of channel uses $\left\{d_{e}+1,\ldots ,d_{U}\right\}$ and incorporates only URLLC transmission. We denote the inputs and outputs of the three parallel channels by

(21a) $$\begin{array}{cc} X_{1}≜\left\{X_{a_{e}},\ldots ,X_{a_{U}-1}\right\}, & \;\;\;\;Y_{1}≜\left\{Y_{a_{e}},\ldots ,Y_{a_{U}-1}\right\}, \end{array}$$

(21b) $$\begin{array}{cc} X_{2}≜\left\{X_{a_{U}},\ldots ,X_{d_{e}}\right\},\;\;\; & \;\;\;\;Y_{2}≜\left\{Y_{a_{U}},\ldots ,Y_{d_{e}}\right\},\; \end{array}$$

(21c) $$\begin{array}{cc} X_{3}≜\left\{X_{d_{e}+1},\ldots ,X_{d_{U}}\right\}, & \;\;\;\;Y_{3}≜\left\{Y_{d_{e}+1},\ldots ,Y_{d_{U}}\right\}. \end{array}$$

For the *i*-th channel with i∈{1,2,3}, the encoding functions satisfy the average block power constraint

(22) $$\frac{1}{n_{i}}\left|\right|X_{i}{\left|\right|}^{2}\leq P_{i}$$

almost surely. The resulting system model is illustrated in Figure 5, where notice that the three channels $P_{Y_{1}|X_{1}}$, $P_{Y_{2}|X_{2}}$ and $P_{Y_{3}|X_{3}}$ are additive, memoryless, stationary, and Gaussian of variances 1.

The scheme further proposes to combine the eMBB and URLLC transmission over the second channel $P_{Y_{2}|X_{2}}$ by means of the simple superposition coding approach described at the end of Section 3.2, for which the block-2 power $P_{2}$ is split into power $\beta P_{2}$ for the eMBB transmission and power $\left(1-\beta_{2}\right)P_{2}$ for the URLLC transmission, for some β∈[0,1]. In particular, the channel inputs $X_{2}$ are formed as

(23) $$X_{2}=X_{2,e}+X_{2,U},$$

where $X_{2,e}$ is a codeword encoding $M^{\left(e\right)}$ of average power $∥X_{2,e}{∥}^{2}=n_{2}\beta P_{2}$, and $X_{2,U}$ is a codeword encoding $M^{\left(U\right)}$ of average power $∥X_{2,U}{∥}^{2}=n_{2}\left(1-\beta \right)P_{2}$.

The Rx first decodes the eMBB message based on the outputs of the first and second channels where it treats the transmission of the URLLC message over the second channel as interference. Subsequently, it decodes the URLLC message based on the outputs of the second and third channel, conditioning on the already decoded eMBB message.

The error probabilities of the described scheme can be analyzed and compared to fundamental lower bounds on the error probabilities, obtained via *meta-converse* arguments [58] with an extension to parallel channels [56]. As shown in [54], the converse and achievability bounds match in specific cases.

**Figure 6:**
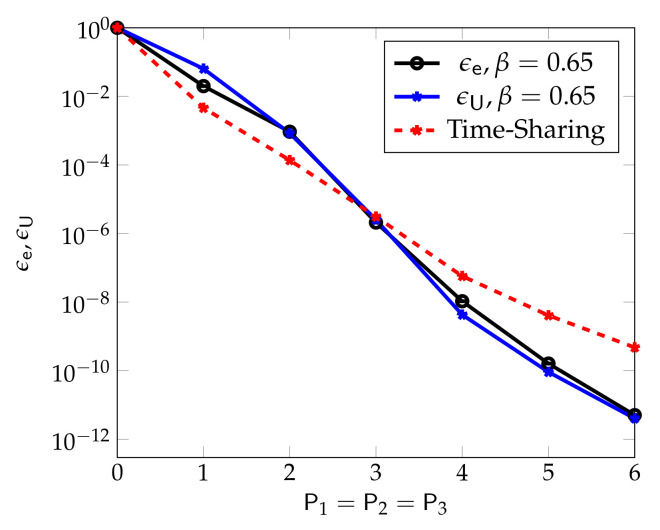
The figure illustrates the average error probabilities of the eMBB and URLLC messages denoted by $\epsilon_{e}$ and $\epsilon_{U}$ in function of the block transmit powers $P_{1}=P_{2}=P_{3}$ and for blocklengths $n_{1}=20,\;n_{2}=20,\;n_{3}=20$, URLLC rate $R_{U}=1/4$, and power split β=0.65.

Figure 6, from [54], compares the performance of the described superposition coding scheme with a standard scheduling scheme that allocates the first half of the channel uses to eMBB transmission and the second half to URLLC transmission. (Under this scheduling approach $\epsilon_{e}=\epsilon_{U}$.) One observes that for the chosen set of parameters, $n_{1}=n_{2}=n_{3}=20$, $R_{U}=1/4$, and β=0.65, the superposition coding approach results in almost identical URLLC and eMBB error probabilities $\epsilon_{e}$ and $\epsilon_{U}$. Moreover, at medium and high powers $P_{2}$, the superposition coding approach significantly outperforms the scheduling approach.

In [55], the authors extend above coding scheme by using the finite-blocklength dirty paper coding (DPC) scheme in [59] to precancel the interference of the eMBB message on the URLLC transmission. Notice that in finite-blocklength DPC, the joint-typicality check is replaced by a norm condition on the input signal, and the Tx has to sacrifice few channel uses to approximately describes the norm of the interference sequence to the Rx. The error probabilities of this DPC based scheme are analyzed in [55] based on the DPC analysis technique in [59] and the parallel channel extension analysis in [56].

**Figure 7:**
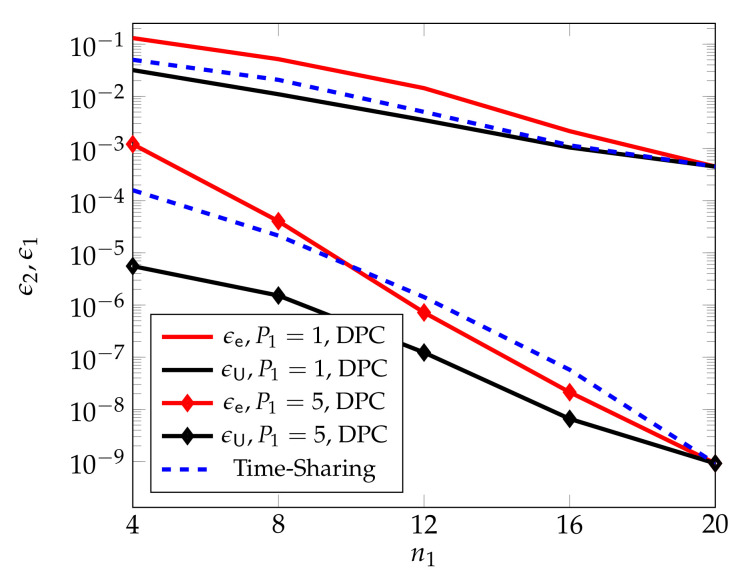
DPC based upper bounds on $\epsilon_{U}$ and $\epsilon_{e}$ in function of the blocklength $n_{1}$ and for average block-powers $P_{1}=P_{2}=P_{3}$.

Figure 7, from [55], compares the performances of the proposed DPC based scheme with standard scheduling, and shows that for large transmit powers $P_{1}=P_{2}=P_{3}$, the DPC based scheme outperforms scheduling over a wide range of blocklengths $n_{1}$.

### 3.4. Summary

This section considered a P2P channel with a single Tx that sends an URLLC message and an eMBB message, where the two types of messages have different decoding delays. In Section 3.2, transmission is over fading channels and URLLC messages have to be transmitted within a single coherence block, whereas eMBB messages can be sent over multiple blocks and thus profit from channel diversity. To compensate for the missing channel state-information at the Tx, an infinite-layer broadcast approach is employed. A closed-form solution for the sum-rate achieved by this broadcast approach was presented, and based on numerical simulations it was observed its maximum sum-rate decreases with increasing URLLC rates. Furthermore, a simplified single-layer power allocation was shown to perform close to the optimal power allocation in the broadcast approach at low URLLC rates.

Section 3.3 studies a related simplified superposition coding or dirty-paper coding schemes but over static Gaussian channels and with fixed creation and decoding times. For this setup, upper and lower bounds on the set of achievable error probability pairs that can simultaneously be achieved for URLLC and eMBB messages was derived in [54]. The obtained results show a performance improvement under these schemes compared to the standard scheduling scheme.

## 4. Broadcast Channels with Mixed-Delay Traffic

### 4.1. Introduction

This section focuses on multi-receiver broadcast channels (BC). The results of this section are based on [60] where similarly to Section 3.2, URLLC messages have to be decoded within a single coherence block but eMBB messages can be transmitted over multiple blocks. In contrast to Section 3.2, the fading powers $\left\{S_{k,b}\right\}$ of the various blocks are known to the various Rxs and the Tx in advance.

### 4.2. Broadcast Approach over Fading Channels

In the setup proposed in [60] each Rx might demand a URLLC message, an eMBB message or both. We thus define the two sets $K^{\left(U\right)}$ and $K^{\left(e\right)}$ indicating the sets of users requesting URLLC and eMBB messages, respectively. Notice that the two sets can overlap. The mixed-delay constraint is captured by imposing a fixed rate on all transmitted URLLC messages, whereas eMBB messages can be either of larger or smaller rates. This rate-adaption on eMBB messages depending on the encountered fading powers enables an increase in the system’s sum-rate. The corresponding optimization problem can be expressed as

(24) $$\max\sum_{k\in K^{\left(U\right)}}R^{\left(U\right)}+\sum_{k\in K^{\left(e\right)}}R_{k}^{\left(e\right)},$$

where the maximization is only over rate-tuples such that URLLC rates ${\left\{R_{k,b}^{\left(U\right)}=R^{\left(U\right)}\right\}}_{k\in K^{\left(U\right)}}$ are achievable on each block b∈{1,…,B} and eMBB rates ${\left\{R_{k}^{\left(e\right)}\right\}}_{k\in K^{\left(e\right)}}$ are simultaneously achievable over the entire transmission, all using dirty-paper coding with an optimal precoding order and under an average block power constraint P.

The optimization problem in (24) is cumbersome to solve, and instead [60] proposes the following suboptimal algorithm. Fix the dirty-paper precoding order to first precode the eMBB messages followed by the URLLC messages. This implies that eMBB transmissions act as noise on the URLLC communication but not vice versa. Then choose a target URLLC rate $R^{\left(U\right)}$ and find the minimum required average block-power βP, for β∈[0,1], that ensures achievability of the per-block and per-user URLLC rate $R^{\left(U\right)}$. Identify finally the maximum sum-rate $\sum_{k\in K^{\left(e\right)}}R_{k}^{\left(e\right)}$ achievable on the eMBB transmission with average power (1−β)P.

Though optimal, dirty-paper coding is difficult to implement in practical systems and is often replaced by the simpler zero-force beamforming. In the context of our multi-user and mixed-delay communication scenario, under zero-force beamforming, it remains to determine the assignment of beams to users and the two communication types. The work in [60] proposes a sophisticated beam assignement algorithm, which assigns stronger sub-channels (beams) to URLLC messages, and weaker channels to eMBB messages. The idea being that eMBB communication can profit from channel diversity over multiple coherence blocks.

Numerical simulations in [60] compare the sum-rate in (24) achieved with dirty-paper coding and with a precoding order and power allocation established according to the suboptimal algorithm described above, with the sum-rate achieved with a beamforming alternative. For both schemes the maximum sum-rate increases with small values of $R^{\left(U\right)}$ and reaches a peak when the URLLC rate contributes approximately a third of the sum-rate. Beyond, the sum-rate decays rapidly because the delay constraint on the URLLC message becomes too stringent and limits the overall performance.

### 4.3. Summary

This section extended the superposition coding approach to fading BCs with multiple Rxs where certain Rxs demand URLLC messages and other eMBB messages. Assuming perfect channel state information, [60] proposes precoding orders or beam assignments for URLLC and eMBB messages, that take into account that URLLC messages have to achieve their desired rates in a single coherence block and therefore cannot exploit the channel diversity offered over multiple blocks. The results in [60] show that for small requested URLLC rates, the sum-rate of the system is not limited by the stringent delay constraint of URLLC messages. For larger URLLC rates this is however the case and URLLC delay constraints limit the overall performance.

## 5. Cooperative Interference Networks

### 5.1. Introduction

In this section we consider interference networks where Txs and/or Rxs can cooperate over dedicated cooperation links. This models for example cellular networks where BSs can cooperate over high-rate fiber-optic links, and neighbouring mobiles can cooperate using bluetooth or millimeter wave communication, which take place on different frequency bands than the standard radio communication between mobiles and BSs, and cause no interference.

**Figure 8:**
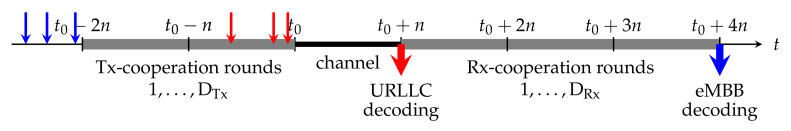
Timeline of cooperation and transmission over the interference network for URLLC and eMBB messages associated to the block from time $t_{0}$ to time $t_{0}+n$.

Cooperation links between Txs are beneficial for eMBB transmissions, because they allow Txs to exchange parts of their messages or their signals so as to enable cooperative signaling over the channel. Cooperation links between Rxs can be used to exchange information about receive signals or decoded messages, allowing the Rxs to better mitigate interference. URLLC transmissions however have to start immediately after the creation of the messages and the additional delays caused by exchanging (parts of) URLLC messages between Txs cannot be tolerated. In the same sense, URLLC messages have to be decoded before Rxs can learn information about other Rxs’ decoded messages or receive signals. Figure 8 illustrates a typical timeline in our model. The actual communication time over the interference network is from time $t_{0}$ to time $t_{0}+n$ and corresponds to the blocklength of communication. Here $t_{0}$ denotes an arbitrary starting time of a block and *n* refers to the block length. It is dedicated to the transmission of URLLC messages generated just prior to $t_{0}$ and of eMBB messages generated prior to $t_{0}-D_{\mathrm{Tx}}\cdot n$, so as to allow the eMBB messages to profit from $D_{\mathrm{Tx}}$ rounds of Tx-cooperation. (For simplicity it is assumed that *n* represents also the length of a cooperation round. The results also extend to scenarios where this is not the case.) Rxs decode the URLLC messages transmitted in this block $\left[t_{0},t_{0}+n\right]$ as soon as the block is terminated, each Rx simply based on its receive signal. Decoding of eMBB messages can be delayed to time $t_{0}+\left(D_{\mathrm{Rx}}+1\right)\cdot n$, until the termination of $D_{\mathrm{Rx}}$ Rx-cooperation rounds.

Consider a scenario where each Tx sends an URLLC message and an eMBB message to its corresponding Rx. The focus is on the set of *degrees of freedom (DoF)* pairs that are simultaneously achievable for URLLC and eMBB messages, and in particular on how the sum-DoF decreases with increasing URLLC DoFs. This decrease describes the degradation of the overall system performance caused by the stringent delay constraints on URLLC messages, as a function of the URLLC rates. Somehow surprisingly, it can be shown that such a degradation does not exist in a variety of networks even with moderate or large URLLC DoFs.

In the following Section 5.2 we describe the problem setup. In Section 5.3, we present the integrated scheduling and coding scheme for URLLC and eMBB messages in [61], and in Section 5.4 we show that this scheme achieves maximum sum-rate even for moderate or large URLLC rates on a variety of network topologies, thus limiting the degradation of the overall system performance. In Section 5.5 we discuss a random-arrival model for URLLC and eMBB messages, where URLLC and eMBB messages are assigned to users according to some random arrival process. Again based on the coding scheme in [61], it can be shown that even under random arrival messages, the overall system performance is hardly degraded by the strict URLLC delay constraints [62,63].

### 5.2. Problem Description

Throughout this section, we consider a cellular network, but assume that users of the same cell are scheduled in different frequency bands. Interference thus occurs only from the mobile users in neighbouring cells that are scheduled on the same frequency band. The system therefore decomposes into subsystems with only a single mobile in each cell.

**Figure 9:**
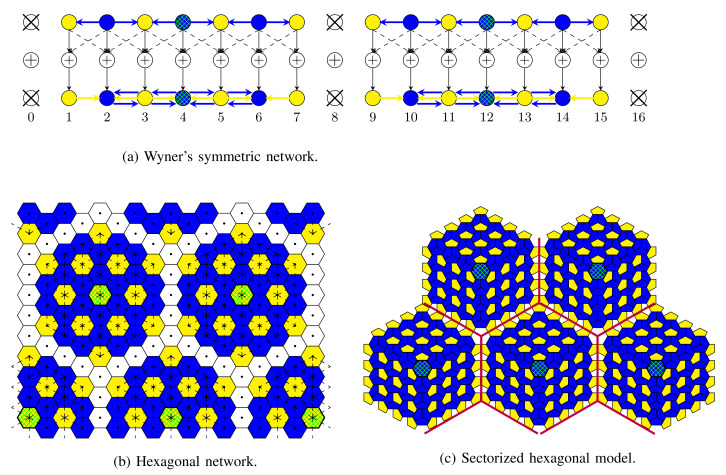
Message assignment for the various network models. White is used for $T_{\mathrm{silent}}$, yellow for $T_{U}$, and blue for $T_{e}$. Master Txs (Rxs) are in green pattern. Maximum number of allowed cooperation rounds D=8.

Consider thus an interference network with K cells, each consisting of a single Tx/Rx pair (i.e., a single mobile/BS pair). Networks have a regular interference pattern except at the network borders, with a focus on three different network topologies with short-range interference:

- Wyner’s linear symmetric model in Figure 9a, where Txs and Rxs are aligned on two parallel lines and interference is only from the two Txs on the left and the right of any given Tx/Rx pair. This topology models for example situations in a corridor or along a railway line or highway where BSs are aligned. Cooperation links are present between neighbouring Txs and between neighbouring Rxs.
- Wyner’s hexagonal model in Figure 9b, where cells are assumed of hexagonal shape. Interference is from the six neighbouring cells. Cooperation links are present between BSs and between mobiles of neighbouring cells.
- Sectorized hexagonal model in Figure 9c, where cells are again of hexagonal shape. In this model, Txs and Rxs use directed antennas, allowing us to divide each cell into three sectors with non-interfering communications, and interference is only from the neighbouring sectors in neighbouring cells, but not from sectors within the same cell. Here, a single mobile user is assumed in each sector, and thus three mobiles in each cell. Cooperation links are present between BSs of neighbouring cells and between mobiles in neighbouring sectors that are not in the same cell.

Each Tx k∈[K] wishes to convey a pair of independent URLLC and eMBB messages $M_{k}^{\left(U\right)}$ and $M_{k}^{\left(e\right)}$ to its corresponding Rx k∈[K]. URLLC Message $M_{k}^{\left(U\right)}$ is of rate $R_{k}^{\left(U\right)}$ and eMBB message $M_{k}^{\left(e\right)}$ of rate $R_{k}^{\left(e\right)}$. The focus is on the average URLLC and eMBB rates

(25) $$\begin{array}{ccc} R^{\left(U\right)} & := & \frac{1}{K}\sum_{k=1}^{K}R_{k}^{\left(U\right)} \end{array}$$

(26) $$\begin{array}{ccc} R^{\left(e\right)} & := & \frac{1}{K}\sum_{k=1}^{K}R_{k}^{\left(e\right)}. \end{array}$$

Consider a cooperation scenario where neighbouring Txs cooperate during $D_{\mathrm{Tx}}>0$ rounds and neighbouring Rxs during $D_{\mathrm{Rx}}>0$ rounds. The total cooperation delay is constrained as

(27) $$D_{\mathrm{Tx}}+D_{\mathrm{Rx}}\leq D,$$

where D≥0 is a given parameter of the system and the values of $D_{\mathrm{Tx}}$ and $D_{\mathrm{Rx}}$ are design parameters and can be chosen arbitrary such that (27) is satisfied. During the $D_{\mathrm{Tx}}$ Tx-cooperation rounds, each Tx can send arbitrary messages to its neighbours depending on the cooperation messages it received in previous rounds and on its eMBB Message $M_{k}^{\left(e\right)}$. In contrast, Tx-cooperation messages cannot depend on URLLC messages, as they are created only shortly before their transmission over the channel. The cooperative communication is assumed noise-free and the total cooperation load over all $D_{\mathrm{Tx}}$ Tx-cooperation rounds on each link is limited to $n\cdot \mu_{\mathrm{Tx}}/2\log\left(1+P\right)$ bits. Each Tx forms then its channel inputs $X_{k}^{n}$ as a function of all its received cooperation messages $T_{k}$ and both its URLLC message $M_{k}^{\left(U\right)}$ and eMBB message $M_{k}^{\left(e\right)}$:

(28) $$\begin{array}{c} X_{k}^{n}=f_{k}^{\left(n\right)}\left(M_{k}^{\left(U\right)},M_{k}^{\left(e\right)},T_{k}\right). \end{array}$$

Channel inputs at each Tx are subject to an average block-power constraint P.

After receiving its channel outputs

(29) $$Y_{k}^{n}=H_{k,k}\;X_{k}^{n}+\sum_{\hat{k}\in I_{k}}H_{\hat{k},k}\;X_{\hat{k}}^{n}+Z_{k}^{n},$$

where $Z_{k}^{n}$ is i.i.d. standard Gaussian noise, matrix $H_{\hat{k},k}$ models the channel from Tx $\hat{k}$ to Rx *k*, which is assumed to be constant during the duration of communication and known by all terminals, each Rx *k* immediately decodes its intended URLLC message:

(30) $${\hat{M}}_{k}^{\left(U\right)}=g_{k}^{\left(n\right)}\left(Y_{k}^{n}\right),$$

using some decoding function $g_{k}^{\left(n\right)}$ on appropriate domains. Following this first decoding step, neighbouring Rxs communicate with each other during $D_{\mathrm{Rx}}$ Rx-cooperation rounds. In each round, each Rx can send arbitrary messages to its neighbours that depend both on the previously received cooperation messages as well as on its output signals. The cooperative communication is assumed noise-free, but its total communication load over all $D_{\mathrm{Rx}}$ Rx-cooperation rounds on each link is restricted to $n\cdot \mu_{\mathrm{Rx}}/2\log\left(1+P\right)$ bits. At the end of these $D_{\mathrm{Rx}}$ Rx-cooperation rounds, each Rx *k* decodes its desired eMBB message as

(31) $${\hat{M}}_{k}^{\left(e\right)}=b_{k}^{\left(n\right)}\left(Y_{k}^{n},Q_{k}\right),$$

where $Q_{k}$ denotes all the Rx-cooperation messages received at Rx *k* and $b_{k}^{\left(n\right)}$ is an appropriate decoding function.

The focus of this section is on the *Degrees of Freedom (DoF) region* of the described model, i.e., on the set of possible pre-log factors $\left(S^{\left(U\right)},S^{\left(e\right)}\right)$ of URLLC and eMBB rates that are simultaneously achievable in the limit of infinite powers P→∞:

(32) $$\begin{array}{ccc} S^{\left(U\right)} & := & \lim_{P\to \infty }\frac{R^{\left(U\right)}\left(P\right)}{\frac{1}{2}\log P} \end{array}$$

(33) $$\begin{array}{ccc} S^{\left(e\right)} & := & \lim_{P\to \infty }\frac{R^{\left(e\right)}\left(P\right)}{\frac{1}{2}\log P}, \end{array}$$

where the pairs $\left(R^{\left(U\right)}\left(P\right),R^{\left(e\right)}\left(P\right)\right)$ need to be simultaneously achievable for given power P.

### 5.3. Coding Schemes

The following coding scheme was presented in [61]. All Txs in the network are scheduled to either send their URLLC message, their eMBB message, or no message at all. The scheduled eMBB and URLLC messages are then jointly transmitted using an integrated URLLC/eMBB coding scheme. Different schedulings can be envisioned to achieve fairness and send all the required messages. Scheduling is described by three sets $T_{\mathrm{silent}}$, $T_{U}$, and $T_{e}$, where

- Txs in $T_{\mathrm{silent}}$ are silenced and Rxs in $T_{\mathrm{silent}}$ do not take any action.
- Txs in $T_{U}$ send only URLLC messages. Tx/Rx pairs in $T_{U}$ are called *URLLC Txs/Rxs*.
- Txs in $T_{e}$ send only eMBB messages. Tx/Rx pairs in $T_{e}$ are called *eMBB Txs/Rxs*.

Figure 9 illustrates the choices of the $T_{\mathrm{silent}}$, $T_{U}$, and $T_{e}$ proposed in [61] for Wyner’s linear symmetric network, the hexagonal model, and the sectorized hexagonal model when the maximum number of allowed cooperation rounds is either D=6 or D=8. White colour is used for Tx/Rx pairs in $T_{\mathrm{silent}}$, yellow colour for pairs in $T_{U}$, and blue colour for pairs in $T_{e}$. The set $T_{U}$ is chosen as large as possible so that URLLC transmissions are interfered only by eMBB transmissions and not by other URLLC transmissions.

Consider the following joint coding scheme, which integrates both eMBB and URLLC messages. eMBB Txs describe quantized versions of their channel input signals during the Tx-conferencing phase to their neighbouring URLLC Txs, which then precancel the interference on their transmissions. URLLC Rxs can thus decode based on interference-free channels. After decoding, URLLC Rxs describe their decoded messages during the Rx-conferencing phase to the adjacent eMBB Rxs, so as to allow them to pre-subtract the interference from URLLC messages before decoding their intended eMBB messages. As a result, with the proposed scheduling and coding, URLLC messages can be decoded based on interference-free outputs and do not disturb the transmission of eMBB messages. For the transmission of eMBB messages, either CoMP transmission or CoMP reception is used, see Section 2.4, but only on subnets. In fact, with the choice of $T_{\mathrm{silent}}$ in Figure 9, the networks decompose into small subnets so that each subnet contains a master Tx/Rx that can be reached by any other Tx/Rx in the subnet with no more than (D−2)/2 hops over the cooperation links. This ensures that CoMP transmission or reception in each subnet is possible with only D−2 cooperation rounds. Since a single cooperation round is used to describe eMBB transmit signals to URLLC Txs and a single round is used to describe the decoded URLLC messages to eMBB Rxs, the scheme respects the maximum number D of total cooperation rounds.

The coding scheme described above transmits both URLLC and eMBB messages. Variants thereof can be used to transmit only eMBB messages or only URLLC messages. More precisely, since any eMBB message can also be treated as a URLLC message (this would mean imposing stringent delay constraints also on some eMBB messages), the same scheme can also be used to send only eMBB messages. An alternative for sending only eMBB messages, is to silence again a set of Tx/Rx pairs and then directly employ CoMP transmission or CoMP reception on the set of non-silenced Txs/Rxs. Both schemes achieve the same DoF, but depending on the specific network they require larger or smaller cooperation rates $\mu_{\mathrm{Tx}}$ and $\mu_{\mathrm{Rx}}$.

A simple way to send only URLLC messages is to choose a largest possible set of non-interfering Tx/Rx pairs and to silence all other Tx/Rx pairs. For Wyner’s linear symmetric network this is optimal. For the two hexagonal models, and for certain channel coefficients, better performance is possible using the interference alignment techniques in [64].

### 5.4. Results on the Joint eMBB/URLLC DoF Region

This subsection presents the achievable eMBB/URLLC DoF region achieved by the schemes in the previous subsection on the three network topologies in Figure 9, and compares them to the outer bounds derived in [61].

First consider Wyner’s linear symmetric network. For this network and for sufficiently large cooperation prelog factors $\mu_{\mathrm{Tx}}$ and $\mu_{\mathrm{Rx}}$, all DoF pairs $\left(S^{\left(U\right)},S^{\left(e\right)}\right)$ in the DoF region are achieved by the schemes described in the previous subsection or by time-sharing different versions thereof. The DoF region is given by the set of all DoF pairs $\left(S^{\left(U\right)},S^{\left(e\right)}\right)$ that satisfy

(34a) $$\begin{array}{ccc} 0\leq S^{\left(U\right)} & \leq & \frac{1}{2}, \end{array}$$

(34b) $$\begin{array}{ccc} 0\leq S^{\left(U\right)}+S^{\left(e\right)} & \leq & \frac{D+1}{D+2}. \end{array}$$

One notices that the sum-DoF of the system is limited by the maximum number of allowed cooperation rounds D. Moreover, the stringent delay constraint on URLLC messages does not penalize the maximum achievable sum-DoF, which is equal to $\frac{D+1}{D+2}$, irrespective of $S^{\left(U\right)}$.

**Figure 10:**
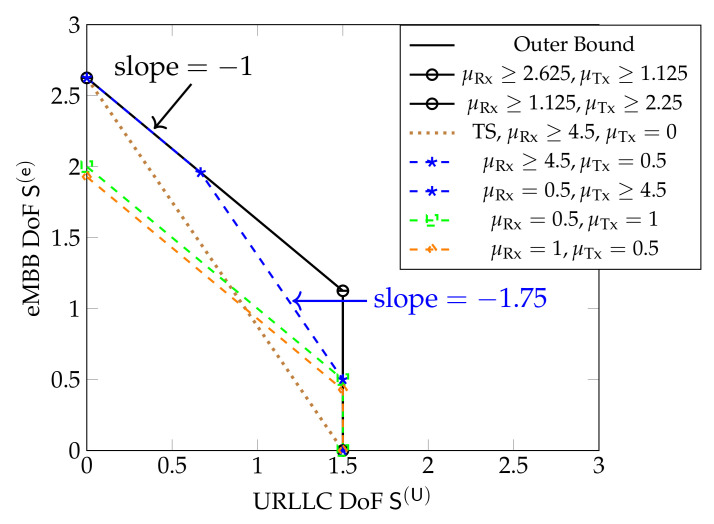
Bounds on DoF region for Wyner’s symmetric model for different values of $\mu_{\mathrm{Rx}}$ and $\mu_{\mathrm{Tx}}$, and D=6. The brown dotted line represents the pure scheduling performance.

For smaller cooperation prelog factors $\mu_{\mathrm{Tx}},\mu_{\mathrm{Rx}}$ this is not the case, as can be seen in Figure 10, which shows inner and outer bounds on the DoF region derived in [61]. The inner bound is achieved by time-sharing the coding schemes in Section 5.3, and significantly improves over a pure scheduling approach that time-shares between a system sending only URLLC messages or only eMBB messages. We notice that for $\mu_{\mathrm{Rx}}\geq 2.625$ and $\mu_{\mathrm{Tx}}\geq 1.125$ the inner and outer bounds match. The inner bound is achieved by the schemes in Section 5.3 employing CoMP reception for eMBB messages. For $\mu_{\mathrm{Rx}}\geq 1.125$ and $\mu_{\mathrm{Tx}}\geq 2.25$ the inner and outer bounds also match, and are achieved by the same schemes, but employing CoMP transmission. When only one of the two cooperation prelogs $\mu_{\mathrm{Tx}}$ or $\mu_{\mathrm{Rx}}$ is large and the other small (e.g., $\mu_{\mathrm{Rx}}\geq 4.5$ and $\mu_{\mathrm{Tx}}=0.5$; or $\mu_{\mathrm{Tx}}\geq 4.5$ and $\mu_{\mathrm{Rx}}=0.5$) the inner bound matches the outer bound only for $S^{\left(U\right)}$ below a given threshold. For URLLC DoFs $S^{\left(U\right)}$ exceeding this threshold, the maximum eMBB DoF $S^{\left(e\right)}$ achieved by the schemes in Section 5.3 decreases linearly with $S^{\left(U\right)}$. For example, for D=6 and $\left(\mu_{\mathrm{Rx}}\geq 4.5,\mu_{\mathrm{Tx}}=0.5\right)$ or $\left(\mu_{\mathrm{Tx}}\geq 4.5,\mu_{\mathrm{Rx}}=0.5\right)$, beyond this threshold, when one increases the URLLC DoF $S^{\left(U\right)}$ by Δ, then the eMBB DoF $S^{\left(e\right)}$ decreases by approximately 1.75Δ and the sum DoF by 0.75Δ. Yet another behavior is observed when both $\mu_{\mathrm{Rx}}$ and $\mu_{\mathrm{Tx}}$ are moderate or small, e.g., $\mu_{\mathrm{Rx}}=0.5$ and $\mu_{\mathrm{Tx}}=1$ or $\mu_{\mathrm{Rx}}=1$ and $\mu_{\mathrm{Tx}}=0.5$. In this case, the sum DoF achieved by the inner bound is not at its maximum value, but constant over all regimes of $S^{\left(U\right)}$. The overall performance of the system is thus again not limited by the stringent delay constraints on URLLC messages, but simply by the available cooperation rates.

**Figure 11:**
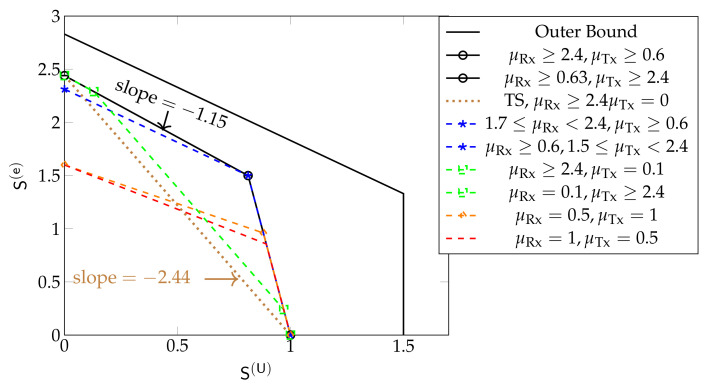
Inner and outer bounds on the DoF region for the hexagonal model for D=8 and different values of $\mu_{\mathrm{Rx}}$ and $\mu_{\mathrm{Tx}}$. The brown dotted line shows the pure time-sharing region.

Figure 11 shows the inner and outer bounds on the DoF region proposed in [61] for the hexagonal model when D=8 and for different values of $\mu_{\mathrm{Rx}}$ and $\mu_{\mathrm{Tx}}$. Unlike in Wyner’s symmetric model, the sum DoF achieved by the schemes in Section 5.3 always decreases as $S^{\left(U\right)}$ increases, irrespective of the cooperation prelogs $\mu_{\mathrm{Tx}},\mu_{\mathrm{Rx}}$. Moreover, the maximum $S^{\left(U\right)}=\frac{L}{3}$ is only achieved for $S^{\left(e\right)}=0$.

**Figure 12:**
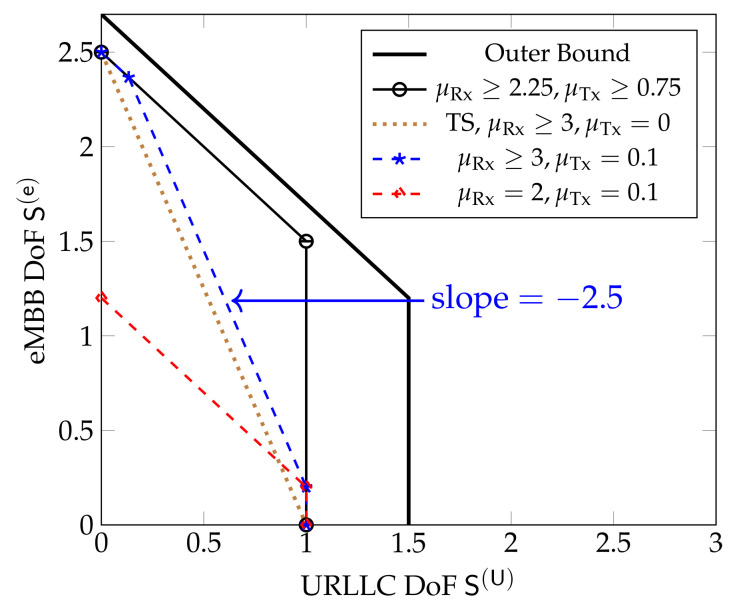
Inner and outer bounds on the DoF region for the sectorized hexagonal model for D=4 and different values of $\mu_{\mathrm{Rx}}$ and $\mu_{\mathrm{Tx}}$. The brown dotted line indicates the pure scheduling performance.

Figure 12 shows inner and outer bounds on the DoF region for the sectorized hexagonal model when D=4. We notice that when both $\mu_{\mathrm{Rx}}$ and $\mu_{\mathrm{Tx}}$ are above given thresholds, $\left(\mu_{\mathrm{Tx}}\geq 0.75,\mu_{\mathrm{Rx}}\geq 2.25\right)$, then the combined scheme integrating both URLLC and eMBB messages in Section 5.3 simultaneously achieves maximum URLLC DoF and maximum sum-DoF. If only one of the two cooperation prelogs is very high but the other one small, the scheme achieves maximum sum-DoF only for small URLLC DoFs. The reason is that the integrated scheme in Section 5.3 that jointly sends URLLC *and* eMBB messages inherently requires both Tx- *and* Rx-cooperation of sufficiently high cooperation prelogs, whereas Tx- *or* Rx-cooperation are sufficient for the scheme that sends only eMBB messages.

To summarize, for all three considered network models the joint scheme in Section 5.3 that integrates both URLLC and eMBB messages achieves maximum sum-DoF at high (or maximum) URLLC DoFs whenever the cooperation rates are sufficiently large. In this case, the stringent delay constraints on the URLLC messages do not harm the overall system performance. For smaller cooperation rates either the maximum sum-DoF is decreased or it is the same as with high cooperation prelogs but can only be achieved for small URLLC DoFs.

The described integrated coding scheme inherently requires at least a single cooperation round both at the Tx-side as well as at the Rx-side. The work in [65] also considered a scenario with only Rx- or only Tx-cooperation. It was shown that when only Rxs or only Txs can cooperate, then the ideal performance in (34) is not possible. Instead for sufficiently large cooperation rates the DoF region is given by the set of all rate-pairs $\left(S^{\left(U\right)},S^{\left(e\right)}\right)$ satisfying [65]

(35a) $$\begin{array}{ccc} 0\leq 2S^{\left(U\right)}+S^{\left(e\right)} & \leq & 1, \end{array}$$

(35b) $$\begin{array}{ccc} 0\leq S^{\left(U\right)}+S^{\left(e\right)} & \leq & \frac{D+1}{D+2}. \end{array}$$

The maximum sum-DoF is thus not decreased compared to a scenario with Tx- *and* Rx-cooperation. However, this maximum sum-DoF is only achievable for URLLC DoF $S^{\left(U\right)}\leq \frac{1}{D+2}$. We conclude that the stringent delay constraint inherently limits the overall system performance for moderate or large URLLC DoFs when only Rxs can cooperate. In fact, in this regime, increasing the URLLC DoF by Δ requires decreasing the eMBB DoF by 2Δ and the sum-DoF by Δ. Similar conclusions also hold for smaller cooperation prelogs and even in the non-asymptotic regime of finite powers [65].

### 5.5. Random User Activities

In practical systems, URLLC messages (and sometimes even eMBB messages) arrive in a random and bursty fashion and consequently in any given block, some Txs do not have an URLLC message to transmit. We consider the *random user-activity and random arrival model* proposed in [62], where each Tx is active with probability ρ, independent of all other Txs. If a Tx is active, it sends an eMBB message to its corresponding Rx, and moreover, with probability $\rho_{f}$, it also sends an additional URLLC message. Both the activity and arrival realizations are assumed to be known to all terminals in the network.

The DoF of *all* URLLC messages in the system is fixed and given by $S^{\left(U\right)}$, whereas the *eMBB* DoF can vary over the variuos eMBB messages, and the quantity of interest is the *expected average* DoF $S^{\left(e\right)}$ over all eMBB messages. (Similarly to the BC scenario in Section 4, the expected average eMBB DoF accounts for the possibility that eMBB messages are sent over multiple URLLC arrival blocks.) The same random-user activity model (but without mixed delays and random message arrivals) was already considered in [66,67,68], where it was observed that under this model the networks considered in Figure 9 decomposes into non-interfering subnets.

**Figure 13:**
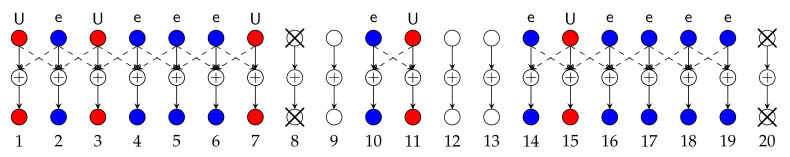
Wyner’s symmetric linear network with random user activity and random arrival. D=6.

The same decomposition happens in the mixed-delay and random message arrivals model. As a consequence, an independent instance of the schemes in Section 5.3 should be applied to each subnet, where the schemes however have to be further adapted to the random URLLC message arrival situation. In particular, the scheduling (choices of sets $T_{\mathrm{silent}},T_{U},T_{e}$) needs to be adapted to the actual URLLC messages present in a subnet. The work in [62] proposes such a new scheduling approach, which on Wyner’s linear symmetric network time-shares between a scheduling that sends URLLC messages at odd Txs and a second scheduling that sends URLLC messages at even Txs. (This allows us to achieve a symmetric URLLC DoF over all users having a URLLC message to send.) The scheduling for odd URLLC Txs is illustrated in Figure 13 for Wyner’s linear symmetric model and specific realizations of the user activities and URLLC message arrivals. In the presented example, Txs 9, 12, 13 are inactive and Txs 1, 3, 7, 11, and 15 have an URLLC message to send. The network thus decomposes into three subnets: the first includes Tx/Rx pairs 1–8, the second includes Tx/Rx pairs 10–11, and the third includes Tx/Rx pairs 14–20. In each subnet, an independent instance of the integrated scheme of Section 5.3 is applied, but where the eMBB message at Tx 19 is treated as URLLC messages to comply with the scheme. In particular, Txs 8 and 20 are silenced because the maximum allowed cooperation delay equals D=8, and Txs/Rxs 4 and 16 act as master Txs/Rxs in the CoMP scheme.

For sufficiently large cooperation rates, the approach in [62] achieves all DoF pairs $\left(S^{\left(U\right)},\;S^{\left(e\right)}\right)$ satisfying

(36) $$\begin{array}{ccc} S^{\left(U\right)} & \leq & \frac{\rho \rho_{f}}{2}, \end{array}$$

(37) $$\begin{array}{ccc} S^{\left(e\right)}+M\cdot S^{\left(U\right)} & \leq & \rho -\frac{\left(1-\rho \right)\rho^{D+2}}{1-\rho^{D+2}}, \end{array}$$

where

(38) $$\begin{array}{c} M≜1+\frac{{\left(1-\rho \right)}^{2}\rho^{D+2}}{\rho \rho_{f}\left(1-\rho^{D+2}\right)}+\frac{{\left(1-\rho \right)}^{2}\rho^{D+1}{\left(1-\rho_{f}\right)}^{\frac{D}{2}}}{\rho \rho_{f}\left(1-\rho^{D+2}{\left(1-\rho_{f}\right)}^{\frac{D}{2}+1}\right)}. \end{array}$$

By means of an information-theoretic converse, it can be shown that all DoF pairs $\left(S^{\left(U\right)},\;S^{\left(e\right)}\right)$ not satisfying (36) and

(39) $$\begin{array}{ccc} S^{\left(e\right)}+S^{\left(U\right)} & \leq & \rho -\frac{\left(1-\rho \right)\rho^{D+2}}{1-\rho^{D+2}} \end{array}$$

cannot lie in the DoF region. Constraints (36) and (39) thus provide an outer bound on the DoF region. Notice that this outer bound and the inner bound given by (36) and (37) only differ in the factors, M>1 or 1, preceding the URLLC DoF $S^{\left(U\right)}$ in the bounds (37) and (39), respectively. These factors are close whenever D≥10, and as a consequence also the presented inner and outer bounds are close. For small values of ρ the factors are already close for D≥4. Moreover, for small values of ρ and D≥4, the right-hand sides of (37) and (39), are approximately equal to ρ, irrespective of D. This indicates that for small values of ρ, increasing the number of cooperation rounds D beyond 4 (and thus further increasing the delay of eMBB messages) does not improve the DoF region of the system. The reason behind this phenomenon is that a large number of cooperation rounds D is only useful in subnets with a large number of consecutive active Txs, and such subnets are very rare when the random user-activity probability ρ is small.

Notice further that by (36), the maximum URLLC DoF both in the inner and outer bounds is $S^{\left(U\right)}=\frac{\rho \rho_{f}}{2}$, because each Tx sends an URLLC message with probability $\rho \rho_{f}$ and in the deterministic setup of Section 5.4, the maximum URLLC DoF is 1/2. Notice also that all bounds (36)–(39) increase with the activity parameter ρ. The maximum eMBB DoF in the inner and outer bounds is $S^{\left(e\right)}=\rho -\frac{\left(1-\rho \right)\rho^{D+2}}{1-\rho^{D+2}}$. In the limit as D→∞, it is thus given by ρ, and simply represents the expected fraction of active users. For finite D, the eMBB DoF decreases because to avoid interference to propagate, some of the Txs have to be silenced as in the scheme of Section 5.3. The term $\frac{\left(1-\rho \right)\rho^{D+2}}{1-\rho^{D+2}}$ thus describes the expected fraction of active but silenced Txs.

**Figure 14:**
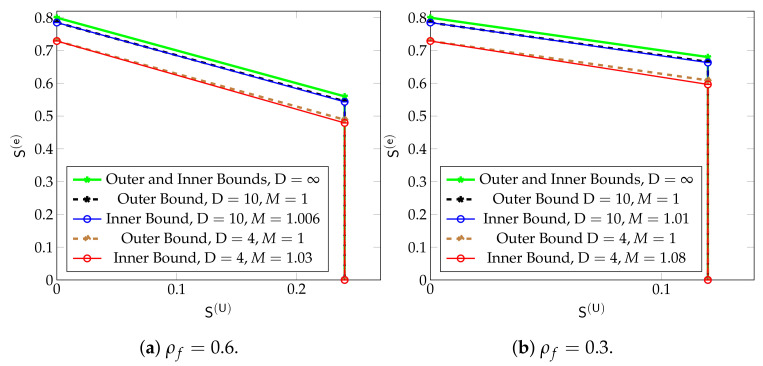
Inner and outer bounds on the DoF region for ρ=0.8 and different values of D.

Figure 14, illustrate the inner and outer bounds in (36)–(39) for different values of $\rho ,\rho_{f}$, and D. The most interesting part of the plots is the upper side of the trapezoids (the side lying opposite the two right angles). The slope of this line, which is −1 for the outer bounds and −M for the inner bounds, describes the penalty in maximum eMBB DoF $S^{\left(e\right)}$ incurred when one increases the URLLC Dof $S^{\left(U\right)}$. Thus, on the outer bounds, increasing $S^{\left(U\right)}$ by Δ decreases the maximum $S^{\left(e\right)}$ by Δ and thus the sum DoF stays constant for all values of $S^{\left(U\right)}$. On the inner bounds, the maximum eMBB DoF $S^{\left(e\right)}$ is decreased by MΔ>Δ when $S^{\left(U\right)}$ is increased by Δ and the sum DoF thus decreases by (M−1)Δ>0.

A similar model, but with only Rx-cooperation was studied in [63] for Wyner’s soft-handoff model, Wyner’s linear symmetric model, and the hexagonal model. Similarly to the setup with deterministic user activities and arrivals, with only Rx-cooperation but no Tx-cooperation the stringent delay requirement on URLLC messages harm the overall performance of the system and maximum sum DoF is only achieved when transmitting only eMBB messages. For networks with regular interference structures as in Figure 9 and when $\rho \rho_{f}\gg 1$, e.g., because URLLC messages are rare, it was shown in [63] that DoF pairs $\left(S^{\left(U\right)},S^{\left(e\right)}\right)$ satisfying the following equality are achievable

(40) $$S^{\left(e\right)}=\rho -\left(1+\ell \rho \right)S^{\left(U\right)},$$

for $S^{\left(U\right)}\leq \frac{\rho \rho_{f}}{\ell }$ and *ℓ* denoting the number of interference signals experienced at each Rx. (For example, for Wyner’s linear symmetric network ℓ=2 and for the hexagonal model ℓ=6).

### 5.6. Summary

This section considered cooperative interference networks where only eMBB messages can profit from these cooperation links, but not URLLC messages because they have to be transmitted and decoded without further due. A general coding scheme was presented that manages to exploit the cooperation links for the transmission of eMBB messages in a way that allows us to attain the optimal overall performance (sum DoF) of the system despite the transmission of URLLC messages. Achieving this performance requires cooperation both at the Tx and Rx side, one of the two is not sufficient. Moreover, a careful scheduling of URLLC and eMBB messages to users had to be performed. In practice this scheduling is performed at a system level in the sense that applications randomly generate URLLC messages. The proposed scheme in [62] was adapted to such random and bursty arrivals of the URLLC messages, with only a small penalty in overall system performance.

## 6. Cloud Radio Access Networks (C-RANs)

### 6.1. Introduction

In this section we consider cloud radio access networks (C-RANs) where BSs are connected to a cloud processor via high-rate fronthaul links and in the uplink communication all transmitted messages are jointly decoded at the central processor so as to be able to alleviate the effect of interference [12]. URLLC messages are however not compatible with this new architecture as they have to be decoded directly at the BSs because communication over an additional hop to the cloud processor would violate their stringent delay constraints. As in the previous sections, we wish to investigate how this restriction affects the overall performance of the systems, and more specifically the sum-rates and rate pairs can simultaneously be achieved for URLLC and eMBB transmissions.

This section reviews two pieces of work on C-RAN with mixed-delay traffic. The work in [69], discussed in Section 6.2 considers a fading network and focuses on an information-theoretic discussion of the problem comparing inner and outer bounds on the fundamental performance limits of such systems. The work in [70,71], discussed in Section 6.3, takes a communication-theoretic approach. It decomposes communication in minislots and then compares performance of different communication strategies. In this latter work, the network is assumed static.

### 6.2. Fading C-RAN Model

**Figure 15:**
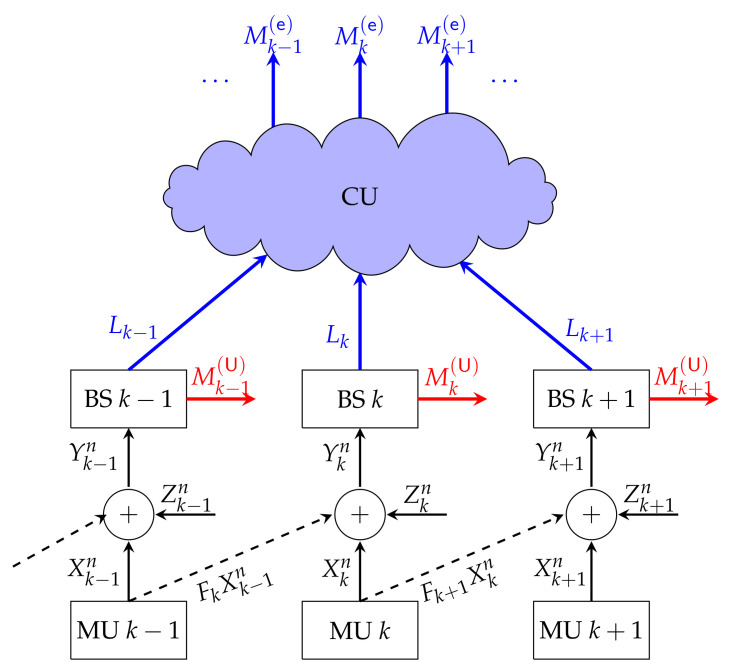
C-RAN with URLLC and eMBB transmissions and the mobile-to-BS network modeled by Wyner’s soft-handoff model.

The work in [69] considers the uplink of a C-RAN, and models the network from the mobile users to the BSs by an i.i.d. fading Wyner soft-handoff model, see Figure 15. That means, BSs are aligned on a line and each cell contains a single mobile user. This latter assumption stems again from the orthogonal frequency access applied by various mobile users in a cell. In Wyner’s soft-handoff model, mobile users are assumed to be located on cell borders and thus interfere only on the communication in this neighbouring and closeby cell. At a given time t∈[n], the signal received at any BS k∈[K] is thus described as

(41) $$Y_{k,t}=G_{k,t}X_{k,t}+F_{k,t}X_{k-1,t}+Z_{k,t},$$

where $X_{k,t}$ and $X_{k-1,t}$ are the signals sent by mobile users *k* and k−1 at time *t*; $\left\{Z_{k,t}\right\}$ are i.i.d standard Gaussian noise; and the sequence of channel coefficients

(42) $${\left\{(G_{1,t},G_{2,t},\ldots ,G_{K,t},F_{1,t},F_{2,t},\ldots ,F_{K,t})\right\}}_{t=1}^{n}$$

is i.i.d. over time and distributed according to a given K-tuple distribution $P_{G_{1}\cdots G_{K}F_{1}\cdots F_{K}}$. This K-tuple distribution is the marginal distribution of a given stationary and ergodic process ${\left\{\left(G_{k},F_{k}\right)\right\}}_{k=-\infty }^{\infty }$ satisfying $E\left[|G_{0}{|}^{2}\right]<\infty$ and $E\left[|F_{0}{|}^{2}\right]<\infty$. The fading coefficients $\left\{\left(G_{k,t},F_{k,t}\right)\right\}$ are known perfectly at BS *k* but not at the mobile users.

Each mobile user *k* sends both an URLLC message $M_{k}^{\left(U\right)}$ and an eMBB message $M_{k}^{\left(e\right)}$. It thus produces its channel inputs as $X_{k}=f_{k}\left(M_{k}^{\left(U\right)},M_{k}^{\left(e\right)}\right)$, and so that they satisfy an average block-power constraint P. URLLC messages are directly decoded at the BSs based on the observed signals in (41). eMBB messages are decoded at the cloud processor, which perfectly observes the symbols sent over the fronthaul links by the BSs, where each BS *k* generates its symbols $L_{k}$ by employing a compression function $f_{k}$ to its observed outputs $Y_{k}^{n}$. The compression has to account for the capacity of the fronthaul links, which is assumed to be $C=\mu \frac{1}{2}\log\left(1+P\right)$ for each link, where μ is termed the fronthaul prelog.

For the described model, ref. [69] characterizes the set of all achievable average expected URLLC and eMBB DoFs (across users and fadings) as

(43a) $$\begin{array}{c} 2S^{\left(U\right)}+S^{\left(e\right)}\leq 1 \end{array}$$

(43b) $$\begin{array}{c} S^{\left(e\right)}\leq \mu . \end{array}$$

The entire DoF region can be achieved by a resource scheduling approach that time-shares URLLC and eMBB transmissions. Notice that the eMBB DoF is limited by the fronthaul prelog μ because all eMBB messages have to be decoded at the cloud center. For small fronthaul prelogs μ this restriction limits the DoF of the system, which can be improved by allowing BSs to also decode part of the eMBB messages.

**Figure 16:**
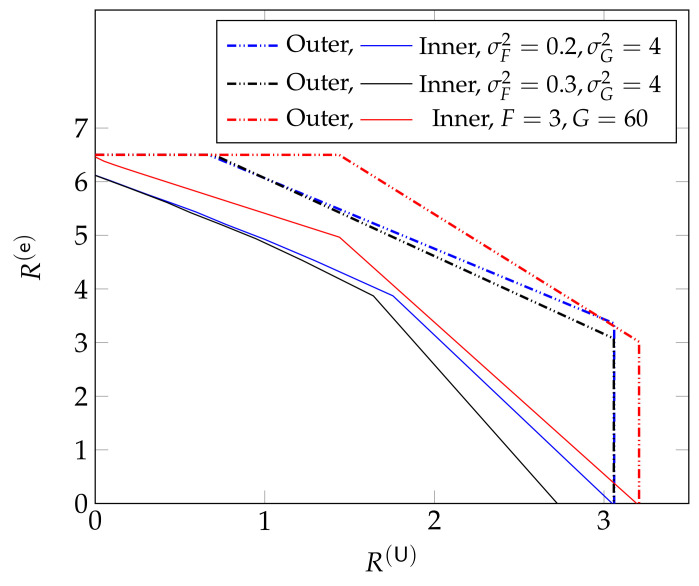
Inner and outer bounds on the achievable $R^{\left(U\right)}$ and $R^{\left(e\right)}$ rate region presented in [69] for P=100, C=6.5, Gaussian i.i.d. fadings of variances $\sigma_{F}^{2}$ and $\sigma_{G}^{2}$ or for constant non-time varying fadings F=3 and G=60.

As can be inferred from (43), the DoF region of the described C-RAN does not depend on the fading processes $\left\{F_{k,t}\right\}$ and $\left\{G_{k,t}\right\}$. This contrasts the behaviour at finite powers P where the set of achievable average URLLC and eMBB rates depends on the law of this fading process, as can be seen in Figure 16. The performance described in this Figure 16 is attained by a superposition coding scheme, and significantly improves over a pure scheduling scheme. We further notice from the figure that for small values of URLLC rates $R^{\left(U\right)}$, when $R^{\left(U\right)}$ increases by Δ, then the maximum eMBB rate $R^{\left(e\right)}$ decreases approximately by the same amount Δ, and thus the sum-rate remains constant. For larger values of $R^{\left(U\right)}$, the maximum $R^{\left(e\right)}$ decreases approximately by 3Δ if $R^{\left(U\right)}$ is increased by Δ. Thereby the loss is larger for random than for static fading coefficients.

### 6.3. Static CRAN Model with Slotted Communication

Mixed delay constraints in C-RANs were also studied in [70], where on a system-wide level communication is divided into minislots. In each minislot, each mobile user generates an URLLC message with probability *q* and attempts to send it over the network during the next minislot that is dedicated to URLLC communication. If a user generates multiple URLLC messages before the next URLLC minislot, it drops all but one URLLC message, which is then sent in this minislot. eMBB messages are sent over multiple minislots and share the available resources in eMBB slots. Moreover, in [70], URLLC communication is assumed to be from mobile users close to the BSs, and their communication does not suffer from intercell-interference. eMBB users are assumed on the network border as in [69] and communication suffers from intercell interference as described by Wyner’s symmetric model.

The performances of different coding schemes are compared in [70]. In all the schemes, eMBB messages are transmitted using standard multi-access codes, since they are jointly decoded at the cloud processor. URLLC messages are transmitted using standard Gaussian codebooks. If eMBB and URLLC messages are sent in the same slots, then eMBB communication (which lasts several minislots) is considered as noise in the decoding of URLLC messages.

- *OMA:* URLLC and eMBB messages are sent using *orthogonal multiaccess (OMA)*, i.e., a pure scheduling approach where the minislots dedicated for URLLC and eMBB communications are disjoint. Specifically, here every $L_{U}$-th minislot is dedicated for URLLC transmission and the other minislots for eMBB transmissions.
- *NOMA–puncturing:* eMBB and URLLC messages are sent using *non-orthogonal multiaccess (NOMA)*, i.e., eMBB and URLLC are sent over the same minislots. In particular, URLLC messages are transmitted in the minislot following their generation. To avoid this URLLC communication interfering with eMBB communication, BSs compress only the signals they receive in minislots where no URLLC communication is taking place from their corresponding mobile user.
- *NOMA–treating URLLC as noise:* eMBB and URLLC messages are sent using NOMA. URLLC communication is treated as noise for eMBB decoding. Therefore, BSs compress all their output signals, and send all this compression information to the cloud processor.
- *NOMA–SIC:* As in the previous item, except that BSs perform *successive interference cancellation (SIC)* on their decoded URLLC messages. That means, if URLLC decoding is successful, they subtract the URLLC signal from their outputs before compressing it for transmission to the cloud processor.

**Figure 17:**
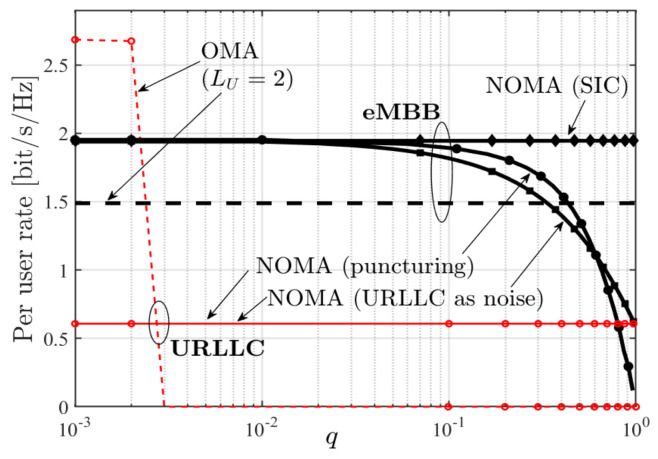
eMBB and URLLC per-user rates under OMA with $L_{U}$ and NOMA for different decoding strategies as function of *q* in C-RAN [70].

The performance of eMBB transmissions is measured by the information-theoretic rate that is achievable in the asymptotic regime of large blocklengths. URLLC transmissions are performed over single minislots and thus of much smaller blocklength. Their performances are measured using a finite blocklength rate-expression [58]

(44) $$R_{U}=\log\left(1+S_{U}\right)-\sqrt{\frac{S_{U}}{n(1+S_{U})}}Q^{-1}\left(\epsilon_{U}\right),$$

where $S_{U}$ denotes the interference power at a BS and $\epsilon_{U}$ has to be chosen sufficiently small so that the overall error probability (including the packet drops in case of OMA) does not exceed a desired threshold. Figure 17 compares the performances of these schemes in function of the URLLC message generation probability *q*. We observe that for the OMA approach performance degenerates quickly even for $L_{U}=2$ because the probability of URLLC message drop is too large. The URLLC performance is identical for all three NOMA approaches. The NOMA SIC approach achieves the best performance for the eMBB message.

In [71], these results are also extended to the downlink scenario. In this case, eMBB messages are created at the cloud processor and can profit from joint encoding to mitigate interference. URLLC messages are created directly at the BSs and their communications thus suffer from interference.

### 6.4. Summary

The last setup considered in this paper are C-RAN architectures where eMBB messages are jointly decoded at the cloud processor, whereas URLLC messages have to be decoded immediately at the BSs. Similarly to the P2P, BC, and cooperative network scenarios, for moderate powers, the overall system performance of the C-RAN with mixed-delay traffic in Section 6.2 decreases for large URLLC rates. This degradation seems to be more pronounced in fading environments than in static Gaussian environments. In the asymptotic high-power regime, however such a degradation is not observed, and at small URLLC DoFs $S^{\left(U\right)}$, the sum-DoF is even increasing in $S^{\left(U\right)}$. In certain scenarios it is thus possible to improve overall system performance by decoding part of the eMBB messages directly at the BSs and not at the cloud processor. Section 6.3 considers a non-fading environment and random generation of URLLC messages, for which it applies finite-blocklength performance measures. It is shown that for moderate or high URLLC generation rates, a NOMA scheme that first subtracts the contribution of the URLLC communication from the receive signals at the BSs, and then compresses and sends these differences over the fronhaul links, outperforms similar OMA and NOMA schemes.

## 7. Conclusions and Outlook

In this survey, we have reviewed joint coding schemes that integrate transmissions of URLLC and eMBB traffic and compared them to pure scheduling schemes in terms of rate, error probability and degree of freedom pairs that the schemes simultaneously achieve for URLLC and eMBB messages. A wide range of communication scenarios including P2P channels, BCs, cooperative interference networks, and C-RANs have been considered. The results have shown that joint coding schemes can significantly outperform the standard scheduling approach. As we have seen, in certain scenarios optimal system performance can be achieved under any URLLC rate. For other scenarios however, a large URLLC rate penalizes the overall system performance, showing that in these situations the stringent URLLC decoding constraint degrades system performance.

We conclude this survey with some lines of potential future research.

- The presented works have considered perfect channel state information (CSI) at the Rxs, and sometimes even at the Txs, where naturally CSI is more difficult to obtain. An interesting model for mixed-delay traffic is where CSI can be used for encoding and decoding of eMBB messages but not of URLLC messages [72,73]. The motivation behind such a model is that the processing of pilot and feedback signals required to gather CSI at the Rxs and Txs introduces inadmissibly large delays for URLLC communication.
- So far, firm finite-blocklength results for mixed-delay traffic have been mostly limited to the P2P case; see [74] for an exception. Extensions to multi-user network scenarios is an important future research direction.
- In practical scenarios, both URLLC and eMBB messages are randomly generated by higher layer applications. This naturally leads to potential bottlenecks where not-yet-transmitted messages have to be buffered, similar to [75]. In this context, a thorough analysis of the behavior of the buffered contents and the required size of these buffers, is of significant practical interest.
- Other heterogeneous requirements on URLLC and eMBB traffic could be introduced in the study of mixed-delay traffic. For example, different security requirements as in [76] or different reliability constraints as in [21].
- Langberg and Effros [77] introduced the notion of *time-rate region* which describes the fraction of the blocklengths required for the transmissions of the various messages to the different Rxs in a network scenario under given message communication rates. A natural question is whether the interference mitigation techniques discussed in this survey can improve the inner bound on the time-rate region for general networks obtained in [77], which is obtained through a reduction to standard network information theory problems.
- Finally, mixed-delay traffic where different messages are transmitted over different blocklengths is inherently also connected to variable-rate and variable-length coding [20,77,78,79]. For example, the variable-rate channel coding framework of [78] includes the variable-to-variable scenario where depending on the specific system configuration and channel state realization, a receiver can decode a message of variable-size (similarly to the broadcast approaches in Sections III-B and IV) and decoding is performed after a variable number of channel uses. An interesting line of future research is to extend this scenario to multiple messages and mixed-delay traffic where URLLC and eMBB messages are decoded with different delays, and to study the four-dimensional tradeoff between URLLC and eMBB variable-rates and variable-delays.

## Figures and Tables

**Table 1:** Related Surveys.

Survey	Year	Comments
[26]	2020	System level perspective on C-RANs.
[11]	2014	System level perspective on C-RANs.
[27]	2019	Overview of network slicing.
[28]	2015	Overview of 5G cellular interference management.
[29]	2019	Machine learning for interference management.
[30]	2022	Survey on rate-splitting in multiple access networks.
[2]	2018	Overview of eMBB and URLLC from a communications theory perspective.
[31]	2018	Survey on control channel design.
[32]	2021	Survey on communication theoretic aspects of 5G.
[33]	2020	Survey on NOMA.
[34]	2019	Survey on NOMA.
[35]	2018	Survey on NOMA.
[36]	2016	Survey on NOMA.

## Data Availability

Not applicable.
